# *RYR1* mutations linked to malignant hyperthermia susceptibility are associated with phenotypic changes in human B-lymphocytes

**DOI:** 10.3389/fimmu.2026.1876709

**Published:** 2026-07-27

**Authors:** Huiying Li, Pamela V. Andrade, Irem Huriye Ceren, Alexis Ruiz, Aline I. Silva, Murilo F. Farias, Thalles S. Zorzeto, Mariz Vainzof, Francesco Zorzato, Helga C. A. Silva, Susan Treves

**Affiliations:** 1 Department of Biomedicine and Neurology, Basel University Hospital, Basel, Switzerland; 2 Brazilian Malignant Hyperthermia Unit. Department of Anesthesiology, Pain and Intensive Care, University Federal of São Paulo, São Paulo, Brazil; 3 Human Genome and Stem Cells Research Center, Institute of Biosciences, University of São Paulo, São Paulo, Brazil; 4 Department of Life Science and Biotechnology, University of Ferrara, Ferrara, Italy

**Keywords:** B-lymphocytes, CD86 and CD40, malignant hyperthermia susceptibility, mutations, ryanodine receptor 1, surface expression

## Abstract

Ryanodine receptor 1 (RyR1) is a key intracellular Ca^2+^ release channel primarily expressed in skeletal muscle, but also in some immune cells. Gain-of-function RYR1 mutations are the most common cause of the pharmacogenetic disorder malignant hyperthermia susceptibility. Compatibly, mice knocked in for gain of function *Ryr1* mutations develop malignant hyperthermia but also show subtle changes in their immune function, supporting a potential but yet to be explored effect of RyR1 mutations on the human immune system. Healthy controls and subjects classified as Malignant Hyperthermia Susceptible were enrolled in an anonymized allergy questionnaire study. PBMC and Epstein Barr virus immortalized B cells from the two cohorts were characterized for surface markers by flow cytometry and for calcium homeostasis using the intracellular calcium indicator Fluo-4. Serum samples were investigated for circulating cytokines by ELISA and Ig quantification was carried out by a clinical laboratory. Malignant hyperthermia susceptible individuals report a higher frequency of allergies, show changes in circulating immunoglobulin and cytokine levels and their circulating B cells exhibit a pre-activated state, with increased surface expression of the co-stimulatory molecules CD86 and CD40. Stimulation of EBV-immortalized B lymphocytes with the RyR1 agonist caffeine results in a tetracaine-sensitive increased surface expression of CD86, which was more pronounced in cells from malignant hyperthermia susceptible individuals. Our results support a functional role of RyR1 in B-lymphocyte Ca^2+^ signaling and show that in humans the presence of gain of function *RYR1* mutations leads to the pre-activation of B cells.

## 1 Introduction

Calcium is a universal second messenger regulating a number of biological functions from muscle contraction, to gene transcription and cell death (1). Ca^2+^ signals result both from the release of Ca^2+^ from intracellular stores (endo/sarcoplasmic reticulum) and influx from the extracellular environment, via the opening of channels on the plasma membrane. In lymphocytes, T-cell receptor and B-cell receptor engagement activate phospholipase C, leading to the production of Inositol 1,4,5 trisphosphate (InsP_3_) and diacylglycerol. InsP3 binds to ligand-gated InsP_3_ receptor Ca^2+^ channels (or InsP_3_R) on the membrane of the endoplasmic reticulum, leading to a rapid and transient release of Ca^2+^ from intracellular stores (2–4). Diacylglycerol activates protein kinase C, which phosphorylates a number of targets, including the transcription factors NFAT and AP-1 (2–4). The breakdown of PIP_2_ via phospholipase C also activates CRAC channels on T cells leading to Ca^2+^ influx from the extracellular environment (3–5). Since in lymphocytes, the endoplasmic reticulum is relatively small, following receptor mediated intracellular Ca^2+^ release, the Ca^2+^ stores are rapidly refilled by a process known as store operated calcium entry (SOCE) via activation of the Stim1/CRAC/Orai1 pathway (3, 5, 6). It is postulated that this SOCE-mediated Ca^2+^ entry is the main activator of Ca^2+^- dependent transcription factors of the calcineurin/NFAT pathway critical for lymphocyte activation, proliferation and survival (7).

Most mammalian cells contain InsP_3_R-gated Ca^2+^ channels, but excitable cells are additionally endowed with ryanodine receptor (RyR) Ca^2+^ channels. In skeletal muscle, RyR1 is an essential player in the process of excitation-contraction coupling, releasing the Ca^2+^ necessary for contraction (8, 9) and mutations in *RYR1*, the gene encoding the RyR1, are associated with a number of neuromuscular disorders including core myopathies, centronuclear myopathy and the pharmacogenetic disorder Malignant Hyperthermia Susceptibility (MHS) (10–12). MHS is triggered by halogenated anesthetics, the muscle relaxant succinylcholine, as well as stress, exercise under excessive heat and viral infections in genetically predisposed individuals most of whom carry heterozygous gain-of-function *RYR1* mutations, causing the RyR1 Ca^2+^ channel to be more sensitive and thus open more easily (12–14). The massive Ca^2+^ release triggered by anesthetics or exogenous triggers, causes metabolic acidosis, muscle rigidity and a rapid increase in body temperature, which if not reversed, can be rapidly fatal (13, 14). MHS is diagnosed either by the *in vitro* contracture test (IVCT), an invasive test requiring a muscle biopsy, or genetically, in patients carrying *RYR1* mutations that have been functionally shown to cause changes in Ca^2+^ homeostasis (13, 14). The calculated frequency of *RYR1* mutations is 1 in 3’000 individuals, and many carriers do not present overt symptoms and are unaware of their genetic status (14, 15).

Though mainly present in skeletal muscle, RyR1 is also expressed in the central nervous system (16), smooth muscle cells (17) and some immune cells (18, 19). Such findings imply that mutations in *RYR1* may give rise to a non-muscle phenotype and indeed, *RYR1* mutations have been associated with enhanced immune functions. For example, (i) mice knocked-in for the MHS-associated *Ryr1* R163C mutation developed more severe symptoms of experimental autoimmune encephalomyelitis than wild type mice (20). (ii) Dendritic cells from mice knocked in for the *Ryr1* Y522S mutation were more efficient at stimulating T cell proliferation; the *Ryr1* Y522S mice also responded faster and more efficiently to antigenic challenges in the early phases of immunization than their control littermates (21). In addition, pharmacological activation of the RyR1 in human dendritic cells in the presence of suboptimal concentration of Toll-like receptor ligands, facilitates their maturation, so that smaller amounts of antigens can bring about effective T-cell responses (19).

However, to date no studies have been performed to investigate how *RYR1* mutations affect the human immune system. In the present study we investigated if and how *RYR1* mutations affect the human immune system by studying a large cohort of Brazilian patients. Our results show that B-lymphocytes from individuals with clinically confirmed MHS express higher surface levels of the co-stimulatory molecules CD86 and CD40 and their serum shows altered levels of circulating cytokines. The results of this study support a role of the RyR1 in B-cell functions and corroborate the hypothesis that gain-of-function *RYR1* mutations linked to MHS are associated with a pre-activated state of B lymphocytes and a potential predisposition to develop specific types of allergies.

## 2 Results

### 2.1 Reported incidence of allergies in the Brazilian MHS population

**Figure 1:**
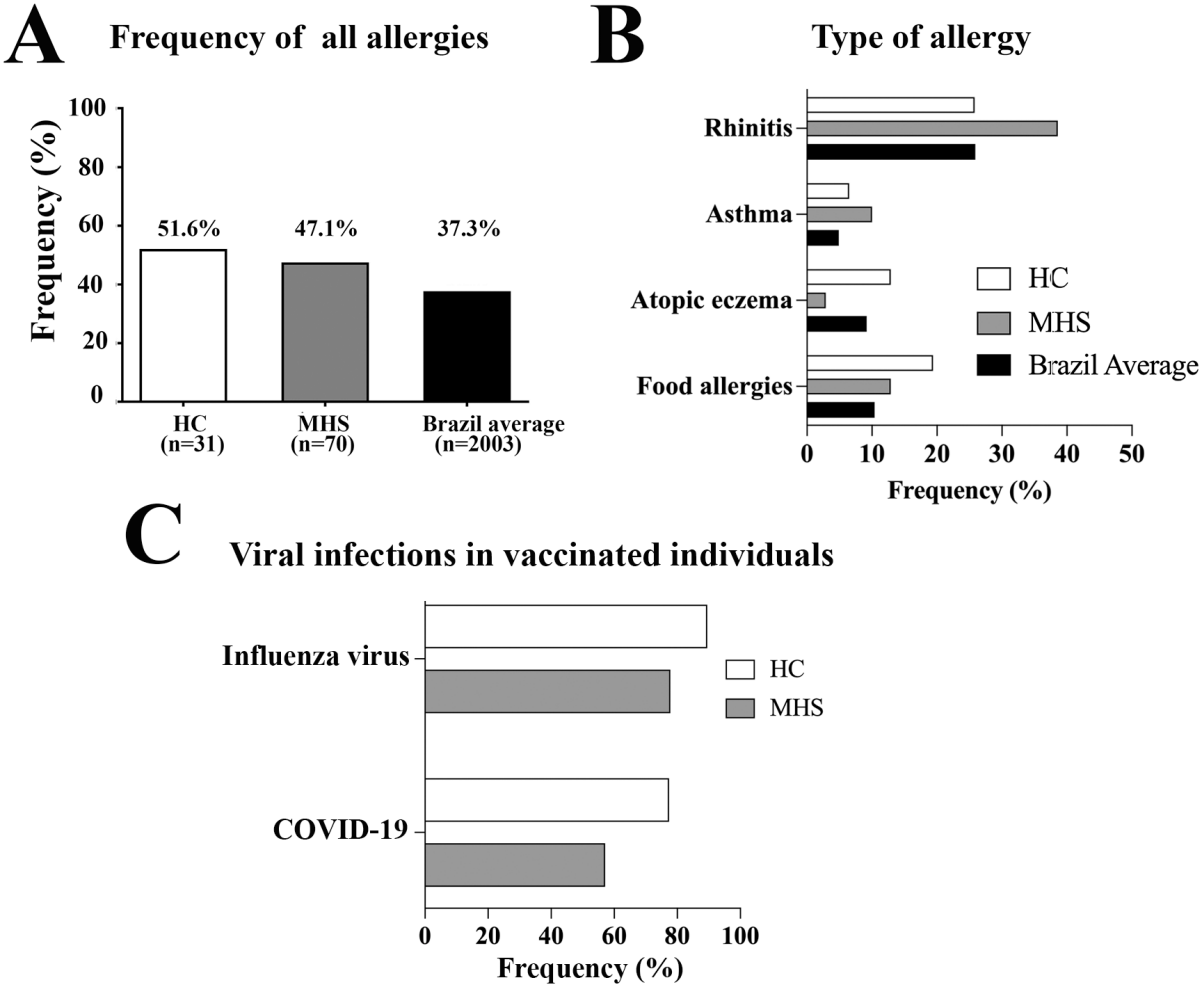
Self-reported incidence of allergic diseases and viral infections in the Brazilian malignant hyperthermia susceptible (MHS) cohort, healthy control (HC) cohort, and a large Brazilian population. **(A)** Bar graph displaying the overall frequency of any reported allergic condition. The white bar represents the healthy control family members (HC, n= 31), the grey bar represents the MHS cohort (n=70), and the black bar represents the results obtained from a published reference on the Brazilian population (n=2’003) (22). **(B)** Bar graph detailing the frequency of specific allergy phenotypes: allergic rhinitis, asthma, atopic eczema and food allergies. White bar = HC, Grey bar = MHS, Black bar = Brazilian population (22). **(C)** Bar graph displaying the frequency of reported Influenza virus and COVID-19 infections in the vaccinated HC cohort (white bars, n=19 for influenza and n=31 for COVID infection, respectively) and MHS cohort (grey bars n=54 for influenza and n=70 for COVID infection, respectively). All graphs are plotted as percentages. No significant differences between MHS and HC when the raw p-values were adjusted using the Benjamini–Hochberg False Discovery Rate (FDR).

Seventy MHS individuals (mean age 45.5 ± 8.8; 51.4% females) plus 24 healthy control (HC) family members and 7 unrelated healthy controls (HC) (mean age 39.5 ± 14.7; 71% females), were enrolled in the allergy questionnaire (**Supplementary Table 1**), which included questions on general health, immunization and allergies. The questionnaire disclosed only one patient with an autoimmune disease (rheumatoid arthritis) and this person only participated in the questionnaire survey. There were no significant differences between the MHS and HC groups regarding age (Fischer’s Exact test), and all enrolled individuals were Brazilian residents recruited in the same way and who completed the same version of the online questionnaire. As far as allergic diseases are concerned, their frequency in the MHS population was 47.1%, with 38.6% reporting allergic rhinitis, 12.9% reporting food allergies, 2.9% reporting eczema and 10% reporting asthma (**Figure 1**; **Supplementary Table 2**). More than one type of allergy occurred in 30.3% of the MHS group; 37 individuals reported that they did not suffer from any allergic condition. The frequency of any allergic disease in the healthy control group was 51.6%, with 25.8% reporting allergic rhinitis, 19.4% reporting food allergies, 12.9% reporting eczema and 6.5% reporting asthma; 15 individuals reported that they did not have any allergic condition. These results show that there are no significant differences between the MHS group and their healthy controls including family members (Fischer’s Exact test) in the overall predisposition to allergies or to specific allergies. However, the small sample size of the healthy control group and the unbalance between the percentage of females and males, motivated us to compare the data of the MHS group to published data on autoimmune disorders in the Brazilian population (22). The total number of subjects investigated in the Brazilian population was 2003 individuals, whose mean age was 38 ± 13.2, and whose sex distribution closely matched that of the Brazilian MHS cohort (58.7% females) (22). There was no significant difference in the overall frequency of reported allergies between the two cohorts (**Figure 1A**). However, the MHS cohort had showed a trend towards a higher frequency of allergic rhinitis (38.6% in MHS vs 25.9% in the Brazilian population; p = 0.026, Fisher’s Exact test) but when more stringent statistical tests were applied to correct for multiple comparisons, the differences were no longer significant (Benjamini-Hochberg adjusted FDR q value =0.129), most likely because of the small number of MHS individuals. In contrast, the frequency of asthma, food allergies and atopic dermatitis/eczema were not significantly different between the two populations (**Figure 1B**; **Supplementary Table 2**).

The same questionnaires also included enquiries about reactions to viral infections such as influenza and COVID-19, as well as reactions to the corresponding vaccines. The COVID- 19 status was confirmed by SARS-CoV-2 Antigen Test or RT-PCR for SARS-CoV-2. The influenza population did not perform specific tests but was based on clinical symptoms/signs and exclusion of COVID-19 infection. Influenza and COVID-19 infection symptoms were classified as follows: +++++ very severe/hospitalization, ++++ severe, +++ similar to other family members, ++ very mild/mild (**Supplementary Table 2**). Regarding influenza virus infection of the vaccinated MHS individuals, 77.8% reported being infected during the year (**Figure 1C**, grey bar), of which 33.3% had very mild/mild symptoms, 9.5% had severe symptoms and 7.1% had very severe symptoms (including 2 individuals who were hospitalized). Of the vaccinated healthy controls, 89.5% reported being infected during the year (**Figure 1C**, white bar), of which 64.7% had very mild/mild symptoms, 5.9% had severe symptoms and none had very severe symptoms (none were hospitalized). Regarding COVID-19 virus infection all individuals were vaccinated, 57.1% of the MHS individuals (**Figure 1C**, grey bar) reported being infected during the year, of which 40% had very mild/mild symptoms, 22.5% had severe symptoms and 7.5% had very severe symptoms (including 3 individuals who were hospitalized). Of the healthy controls, 77.4% reported being infected during the year (**Figure 1C**, white bar), of which 41.7% had very mild/mild symptoms, 16.7% had severe symptoms and 4.2% had very severe symptoms (including 1 that was hospitalized). One individual could not remember the severity of his infection. As far as reported adverse reactions to the influenza vaccine and COVID-19 vaccine administration, there were no significant differences between the MHS and healthy control groups (**Supplementary Table 2**).

In conclusion, the questionnaire study raises the interesting observation that MHS individuals report a higher frequency of allergic rhinitis and may be less susceptible to viral infections, a potential consequence of carrying mutant RyR1 calcium channels in their immune cells.

### 2.2 Characterization of circulating peripheral blood leucocytes and serum of MHS and healthy controls

**Figure 2:**
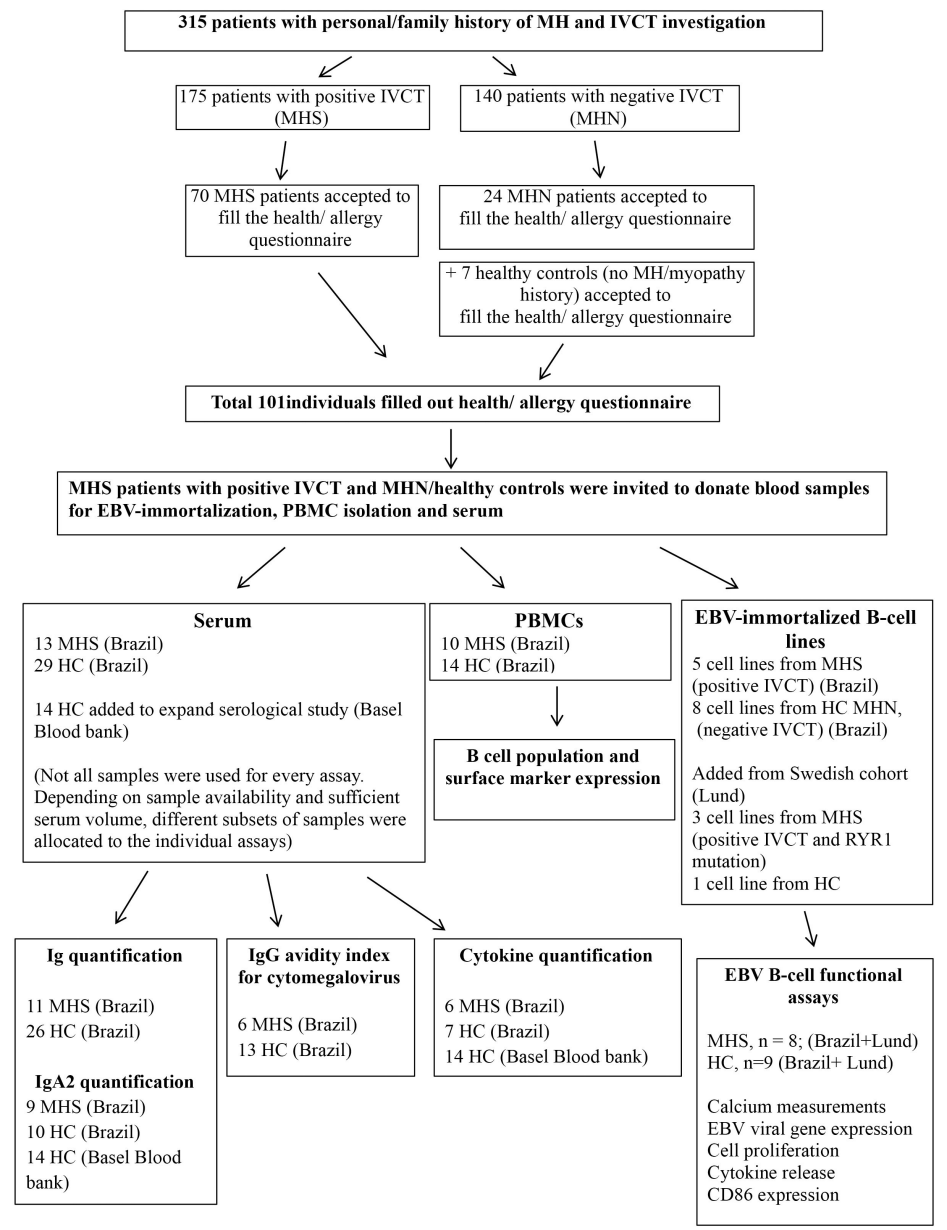
CONSORT diagram, illustrating participant enrollment, health/allergy information and biological sample collection of participants enrolled in the present investigation.

In order to gain insight into the mechanism responsible for the possible enhanced immune functions of the MHS cohort, we investigated peripheral blood leukocytes and serum samples from the Brazilian cohort including 29 healthy controls (HC) and 13 individuals whose MHS status was confirmed by the *in vitro contracture test* (IVCT). The IVCT test results and *RYR1* mutations carried by the MHS individuals are listed in **Supplementary Table 3**. Of note, not all individuals donating PBMCs and serum answered the allergy questionnaire and not all blood donors could be enrolled in all the biological assays because of the limited amounts of blood that could be withdrawn from each individual. All blood donors were free of viral infections, did not exercise prior to blood donation, did not show any acute symptoms correlated to their MHS status and the concentration of C-reactive protein (CRP) was below the detection level in all the serum samples, ensuring that any observed differences between the HC and MHS cohort could not be ascribed to an underlying infection or inflammation. **Figure 2** summarizes the work flow in the form of a CONSORT diagram, illustrating participant enrollment, health/allergy information and biological sample collection.

**Figure 3:**
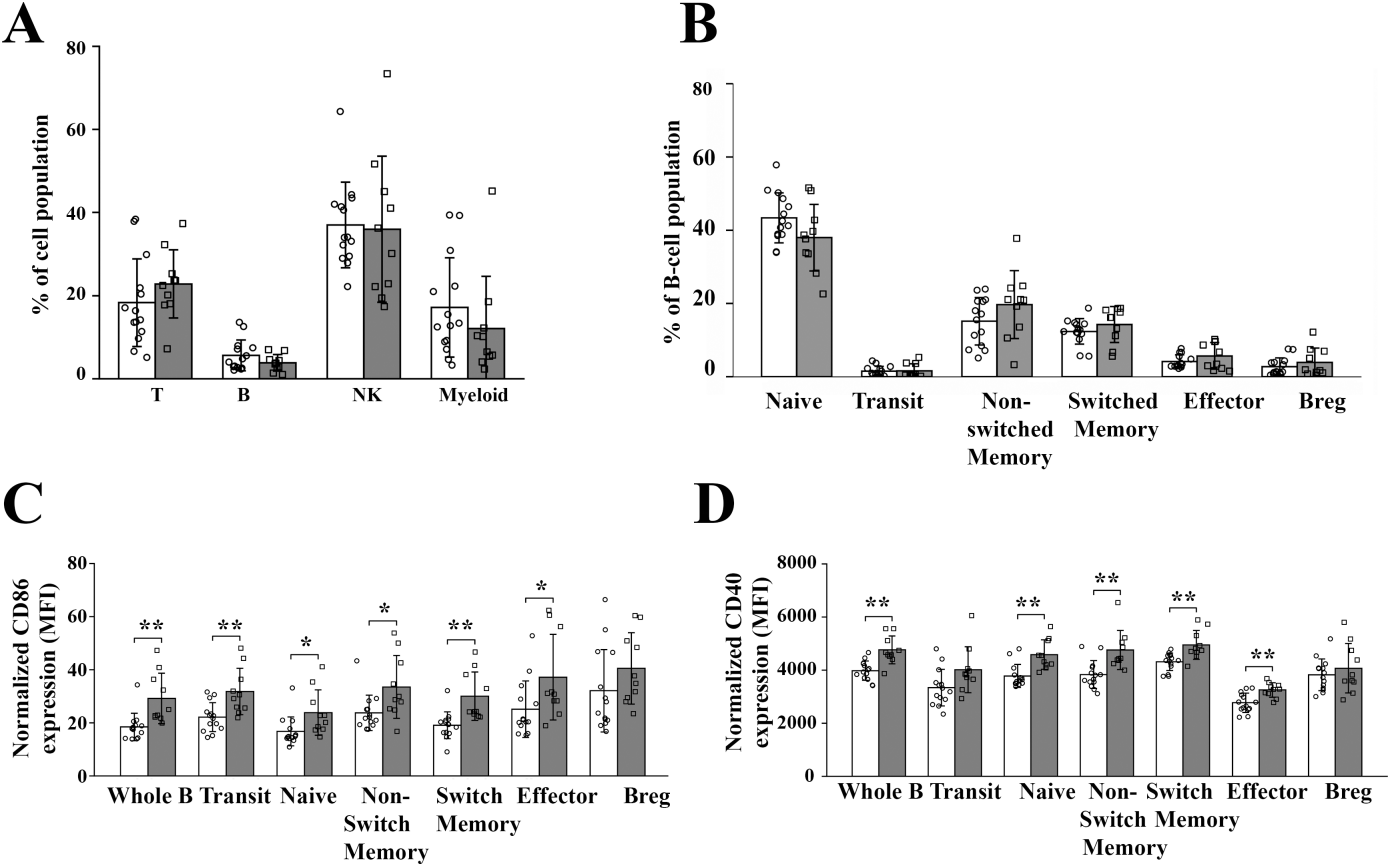
The surface expression of CD86 and CD40 are increased in circulating CD19^+^ B cells from MHS relative to healthy controls. **(A)** Bar graph displaying the percentage of major circulating immune cells (T cells, B cells, NK cells and myeloid cells) in PBMCs from healthy controls (HC) and MHS individuals. **(B)** Bar graph showing the relative frequencies of specific B-cell subpopulations in PBMCs. **(C)** Bar graph showing the relative Mean Fluorescence Intensity (MFI) of surface CD86 in the total B-cell population and within specific B-cell subsets. **(D)** Bar graph showing the relative MFI of surface CD40 in the total B-cell population and within specific B-cell subsets. All data are expressed as mean (± s.d.). White bars, HC cohort (n = 14), grey bars, MHS cohort (n = 10). Statistical comparisons between the HC and MHS cohorts for panel A and B were performed using Welch’s t test. No significant differences were found between HC and MHS in the % major circulating leukocytes [panel **(A)**] or % B cell subset distribution [panel **(B)**]. For panels **(C, D)**, involving multiple simultaneous comparisons of surface markers across B-cell subsets, raw p-values were adjusted using the Benjamini–Hochberg False Discovery Rate (FDR) and are reported as q-values when statistically significant. Statistical significance was defined as *q<0.05 and **q<0.01.

We first investigated whether the presence of *RYR1* mutations impacts the frequency of the major circulating immune cells (**Figure 3A**). The relative percentage of T and B lymphocytes, NK cells and myeloid cells was not different between HC (white bars, n=14) and MHS (grey bars, n=10). A detailed analysis of the B-cell subpopulations in PBMCs showed similar frequencies of naïve, transitional, non-switched memory, switched memory, effector, and regulatory B cells (**Figure 3B**) (see Supplementary Supporting data for all mean ± s.d.). We next verified whether in the human MHS cohort, the presence of *RYR1* mutations leads to changes in the surface expression of specific maturation markers which could influence their capacity to present antigens to T cells via enhanced expression of co-stimulatory molecules. Indeed, in a previous study on the immune system of *Ryr1* Y522S mice, we showed that antigen-presenting CD11c^+^ dendritic cells from the knock-in mice, had a significant increase in the surface expression of the maturation marker CD83 (21). We examined different surface markers across the B cell subsets of PBMCs from 10 MHS and 14 HC, including activation markers (CD86, CD83, MHC-II, CD40, CD71 and BAFFR), regulatory markers (PD-L1, CD39 and CD85j) and surface Ig (including IgM, IgD, IgG and IgA) (for detailed gating strategy see **Supplementary Figures 1A, B**). Interestingly, the surface expression of CD86 within specific B cell populations was significantly increased in the MHS cohort (grey bar, n=10) compared to healthy controls (white bar, n=14) (**Figure 3C**). Indeed, CD27^−^IgD^+^CD10^+^ transitional B cells (Benjamini-Hochberg adjusted FDR q=0.0069), CD27^-^IgD^+^CD10^-^ naïve B cells (Benjamini-Hochberg adjusted FDR q=0.029), CD27^+^IgD^+^ non-switched memory (Benjamini-Hochberg adjusted FDR q=0.029), CD27^+^IgD^−^ switched memory (Benjamini-Hochberg adjusted FDR q=0.006) and CD38^-^CD21^-^ effector B cells (Benjamini-Hochberg adjusted FDR q=0.0443), all showed increased CD86 surface expression (see Supplementary Supporting data for all mean ± s.d.). A significant increase in CD40 surface expression was also observed in B cell subpopulation of MHS individuals (**Figure 3D**). Specifically, within B cell subsets, CD27^-^IgD^+^CD10^-^ naïve B cells (Benjamini-Hochberg adjusted FDR q=0.0022), CD27^+^IgD^+^ non-switched memory (Benjamini-Hochberg adjusted FDR q=0.0024), CD27^+^IgD^−^ switched memory (Benjamini-Hochberg adjusted FDR q=0.0024) and CD38^-^CD21^-^effector B cells (Benjamini-Hochberg adjusted FDR q=0.0024), all showed increased CD40 surface expression. For representative overlay histograms see **Supplementary Figures 1C, D** and see Supplementary Supporting data file for all mean ± s.d. and exact q values. No significant changes in expression of other activation surface markers, including MHC-II, CD83, CD71 and BAFFR were observed in the B-cell populations between MHS and HC cohorts (**Supplementary Figure 2**). We also verified whether the increased surface expression of maturation markers in cells from the MHS is common to other cell types or specific to B cells. To this end, we assessed surface expression of CD86, CD40, CD83 and MHC-II in the myeloid cell compartment (CD45+ CD56- MHC-II^high^ CD14+) from the HC and MHS individuals. No difference was observed between the two cohorts (**Supplementary Figure 3**), substantiating that in humans, the increased surface expression of the maturation markers CD86 and CD40 is limited to B-cells and not a unifying effect on antigen presenting cells.

**Figure 4:**
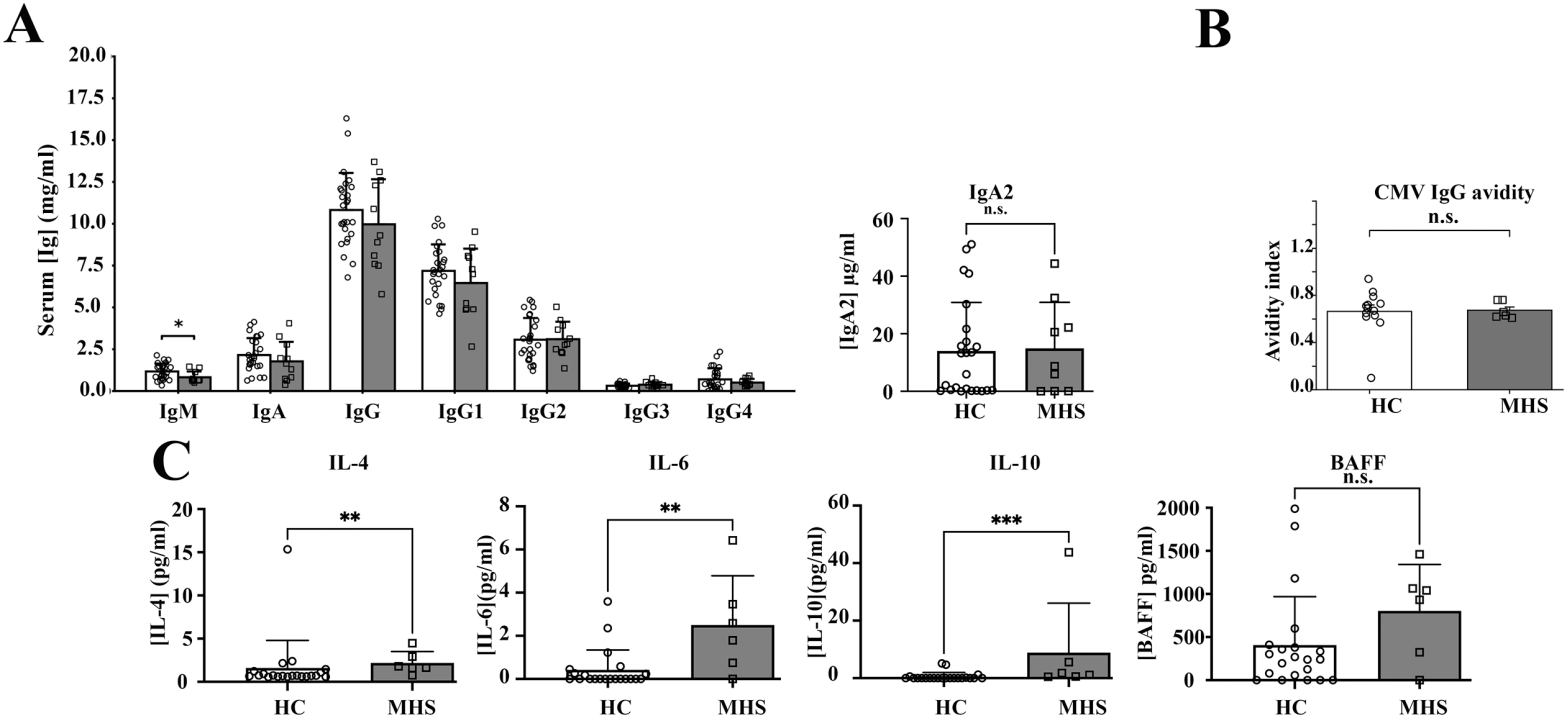
Serum samples from MHS individuals show increased levels of IL-4, IL-6 and IL-10. **(A)** Left panel: Ig concentration in the serum samples from healthy controls (HC, n=26) and malignant hyperthermia susceptible individuals (MHS, n=11). Bar graphs show the mean (± s.d.) concentration of the indicated Ig. Right panel. Mean IgA_2_ concentration in human sera. Individual data points represent serum samples from HC (n= 24) and MHS individuals (n=9). ELISA experiments were performed in technical duplicates. **(B)** IgG avidity index for cytomegalovirus in serum samples from HC (n= 13) and MHS individuals (n=6). **(C)** Serum concentration of IL-4, IL-6, IL-10 and BAFF. HC (n= 21), MHS individuals (n=6). ELISA experiments were performed in technical duplicates. White bars, HC, grey bars MHS. Bars represent the mean (± s.d.) and each symbol represents results from one individual. Statistical significance was determined using Welch’s t test for IgM levels *p<0.05 and Mann-Whitney U test for cytokines **p< 0.01, ***p< 0.001. n.s. means not significant.

Next serum samples from the same group of MHS (n= 11) and HC individuals (n=26) were investigated for changes in circulating levels of immunoglobulins and, when sufficient amounts of serum were available, for circulating cytokines. Interestingly, the MHS group (**Figure 4A**, grey bars) showed lower levels of circulating IgM compared to healthy controls (**Figure 4A** white bars). The concentration of IgM was 0.84 ± 0.34 mg/ml vs 1.18 ± 0.47 mg/ml in MHS vs HC, respectively (p=0.022 Welch’s t test) whereas the concentration of all other Ig classes and sub-classes including IgA2 was similar between the two groups (**Figure 4A**) (see Supplementary Supporting data for all mean ± s.d and exact p values). We also investigated whether the MHS phenotype correlated with changes in immunoglobulin avidity, as this would indicate enhanced B-cell immunocompetence, by assessing the avidity index of IgG for cytomegalovirus (CMV), a viral infection which is common in the general population. In this case, the serum of 6 out of 8 MHS and 13 out of 17 HC had detectable IgG but there was no change in the avidity index of IgG for CMV antigen (**Figure 4**).

Since the number of serum samples from the healthy control group was relatively small, for the analysis of circulating cytokines we expanded the HC cohort from 7 to 21 by adding serum samples from healthy donors collected from the local blood bank (Blutspende SRK Nordwestschweiz, Basel, Switzerland). The amount of serum available from all MHS patients was not sufficient, and we could only analyze cytokine content from 6 MHS individuals (**Supplementary Table 3**). We evaluated the levels of circulating cytokines, focusing on those playing a role in inflammation, allergies and B-cell function, including IL-1ß, IL-2, IL-4, TNF-α, IL-6, IL-10, IL-12 and BAFF. All samples had detectable levels of IL-4 and its concentration was significantly higher in MHS (grey bar) than HC samples (white bar) (p=0.0063 Mann-Whitney U test.). Zero out of 6 MHS and 18/21 HC had undetectable levels of IL-6, and the MHS (grey bar) samples had higher concentrations than HC (white bar) (p=0.005 Mann-Whitney U test). Zero out of 6 MHS and 18/21 HC had undetectable levels of IL-10 and MHS (grey bar) samples had higher levels than HC (white bar) (p=0.0004 Mann-Whitney U test) (**Figure 4**; **Supplementary Figure 4** for heat map). The concentration of BAFF was also higher in the MHS group; 1/6 MHS and 5/21 HC had undetectable levels but due to the great interpersonal variability, these differences were not statistically significant (**Figure 4**) (see Supplementary Supporting data for all mean ± s.d. and exact p values). The concentration of the other cytokines (IL-1ß, IL-2, TNF-α and IL-12) was below the detection level in the sera of the HC and MHS cohorts.

Since all MHS serum samples were obtained from the Brazilian cohort, while control samples originated from both Brazil and Basel, a potential site effect was assessed within the healthy control group by comparing IgA2 and cytokine levels between the two control cohorts. **Supplementary Figure 5** shows that the levels of the cytokines and IgA2 were similar between the Brazilian and Basel blood bank cohorts substantiating our finding that the differences in IL-4, IL-6 and IL-10 observed between the MHS group and the HC group are unlikely due to differences in sample origin (Brazil versus Basel).

In conclusion, analysis of the circulating immune cells of MHS and HC revealed that the presence of *RYR1* mutations does not cause any changes in the relative proportion of circulating leukocyte types, nor in the sub-types of CD19+ve B-cells. However, B lymphocytes from MHS individuals show significantly higher levels of surface expression of the co-stimulatory molecules CD86 and CD40, MHS have lower serum levels of IgM and increased levels of IL-4, IL-6 and IL-10 supporting the hypothesis that the presence of *RYR1* mutations leads to subtle changes in the immune system.

### 2.3 Characterization of EBV-immortalized B cells from HC and MHS individuals

**Figure 5:**
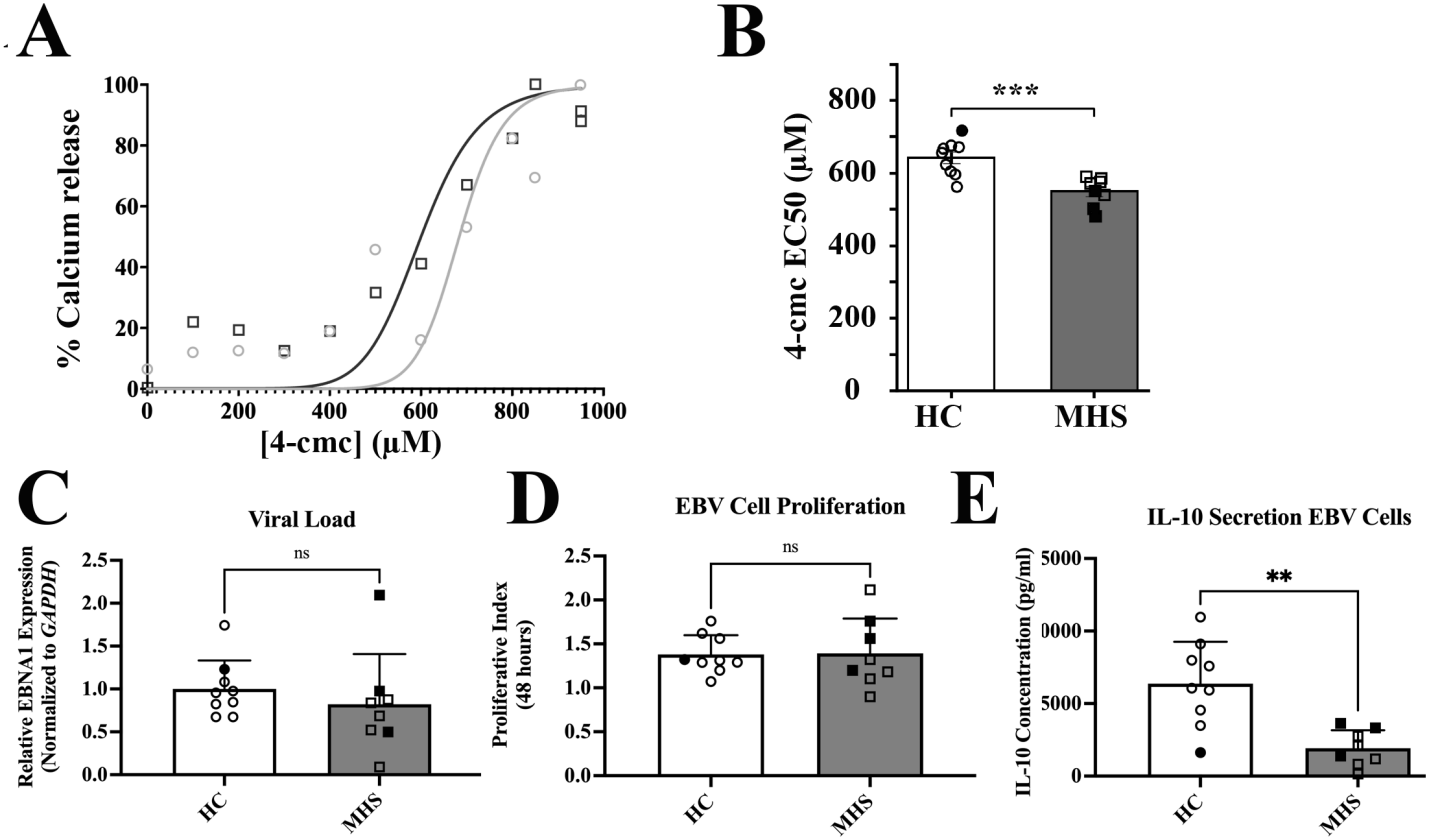
Characteristics of EBV-immortalized B cells from HC and MHS: **(A)** Representative 4-chloro-m-cresol (4-cmc) dose response curve. For each 4-cmc concentration, the ΔFluo-4 fluorescence (change in cytosolic calcium), was calculated as peak Fluo-4 fluorescence-resting fluorescence in arbitrary units, and is plotted as a % of the maximal ΔF obtained at 900 µM 4-cmc and used to generate the dose response curves. Light grey line and circles, healthy family member (HC9); dark grey line and squares, Malignant Hyperthermia Susceptible individual (MHS3). **(B)** EC_50_ for 4-cmc induced calcium release. 4-cmc concentration (µM). For each cell line triplicate experiments were performed as detailed in the Methods section. **(C)** Relative gene expression of the EBV viral gene *EBNA1* in HC and MHS-derived cell lines. Each data point represents an individual cell line, reflecting the mean of at least three independent qPCR experiments performed in technical duplicates. *EBNA1* expression was normalized to the internal housekeeping gene *GAPDH*. **(D)** Proliferation index of EBV-immortalized cell lines from HC and MHS individuals. The proliferation index was calculated as the ratio of the absorbance (490 nm) at 48 hours to that at time 0 (OD_T48/_OD_T0_) following an MTS based assay. Each data point represents the mean of at least three independent measurements performed in technical duplicates. **(E)** Concentration of IL-10 released into the culture supernatant by EBV-immortalized cells from HC and MHS individuals. Cells were incubated for 16 hours at 37 °C prior to supernatant collection. Each data point is the mean of at least three independent experiments performed in technical duplicates. For all panels, white bars, HC (each circle represents one individual n=9), grey bars, MHS (each square represents one individual n=8). Empty symbols, Brazilian cohort, filled symbols, Swedish cohort. All data are presented as mean (± s.d.) Statistical significance was determined using Mann-Whitney U test **p< 0.01, ***p< 0.001. n.s. means not significant.

The results obtained so far show that the MHS phenotype is parallelled by changes in the surface expression of CD86 and CD40, however they do not directly demonstrate that it is the activation of RyR1-mediated calcium release that is responsible for these changes. In order to study directly the effect of *RYR1* mutations on B cell function, we immortalized B-lymphocytes from MHS individuals with a positive IVCT as well as cells from healthy controls (see **Supplementary Table 3** for patient genotype and IVCT result). EBV-immortalized B-lymphocytes express the RyR1 and have been used as a model to study the functional effects of mutations in *RYR1* identified in human patients (23, 24). To confirm that the EBV-immortalized B cells from the Brazilian MHS cohort are more sensitive to the pharmacological activation of the RyR1, we assessed the sensitivity of Ca^2+^ release following stimulation with the RyR1 agonist 4-chloro-m-cresol (4-cmc) (25). Cells from MHS and HC were loaded with the fluorescent calcium indicator Fluo-4 AM, and baseline fluorescence was recorded before the addition of 4-cmc. Subsequently, different concentrations of 4-cmc, ranging from 100 µM to 950 µM, were added to the cells, the changes in fluorescence were monitored using a fluorescence plate reader and dose response curves to 4-cmc were generated. **Figure 5A** shows a representative dose response curve of EBV-immortalized B-lymphocytes from MHS3 carrying the p.I2453T RyR1 mutation (dark grey line) and his HC family member (HC9, light grey line), confirming that the presence of a *RYR1* mutation causes a shift in the sensitivity to 4-cmc. In order to expand the number of cells to be investigated in the next section, we also included EBV-immortalized B-lymphocytes from a genetically characterized Swedish cohort (26), bringing the total number of EBV-immortalized cell lines to 9 HC and 8 MHS (**Supplementary Table 3**). **Figure 5B** shows that cells from the MHS group (grey bar) have a significantly lower 4-cmc EC_50_ than cells from the healthy control group (white bar) (p= 0.001 Mann-Whitney U test; see Supplementary Supporting data for all mean ± s.d and exact p values).

We next studied whether there are basic differences between the immortalized B cell lines from the HC (white bars) and MHS (grey bars) individuals. There were no differences in viral load (**Figure 5C**), nor in the proliferative capacity (**Figure 5D**) of the cell lines obtained from the two groups. Secreted and intracellular levels of IL-2, IL-4, IL-6, IL-1α, IL-1β and TNF-α were undetectable in all EBV-immortalized B-lymphocytes, while IL-12 and IL-10 were successfully detected and quantified. While there were no significant differences in the intracellular concentrations of IL-12 and IL-10 (**Supplementary Figure 6**), the concentration of secreted IL-10 was significantly lower in the EBV-immortalized B cells from the MHS group (grey bar) compared to HC (white bar) (**Figure 5E**). The mean ± s.d. concentration of IL-10 released during 16 hours of incubation was 1914.11 ± 1242.27 pg/ml/10^6^ cells in the MHS-derived cells and 6362.75 ± 2898.43 pg/ml/10^6^ cells in HC-derived cells and (p= 0.002, Mann-Whitney U test).

In conclusion EBV-immortalized B cells from MHS individuals are more sensitive than those from HC to pharmacological ryanodine receptor stimulation, releasing larger amounts of calcium at lower agonist concentration. MHS cells also spontaneously released lower amounts of IL-10 compared to cells from HC, suggesting that the release of this cytokine is influenced by intracellular calcium homeostasis in immortalized B lymphocytes.

### 2.4 Stimulation of EBV-immortalized B cells with caffeine induces surface expression of CD86

**Figure 6:**
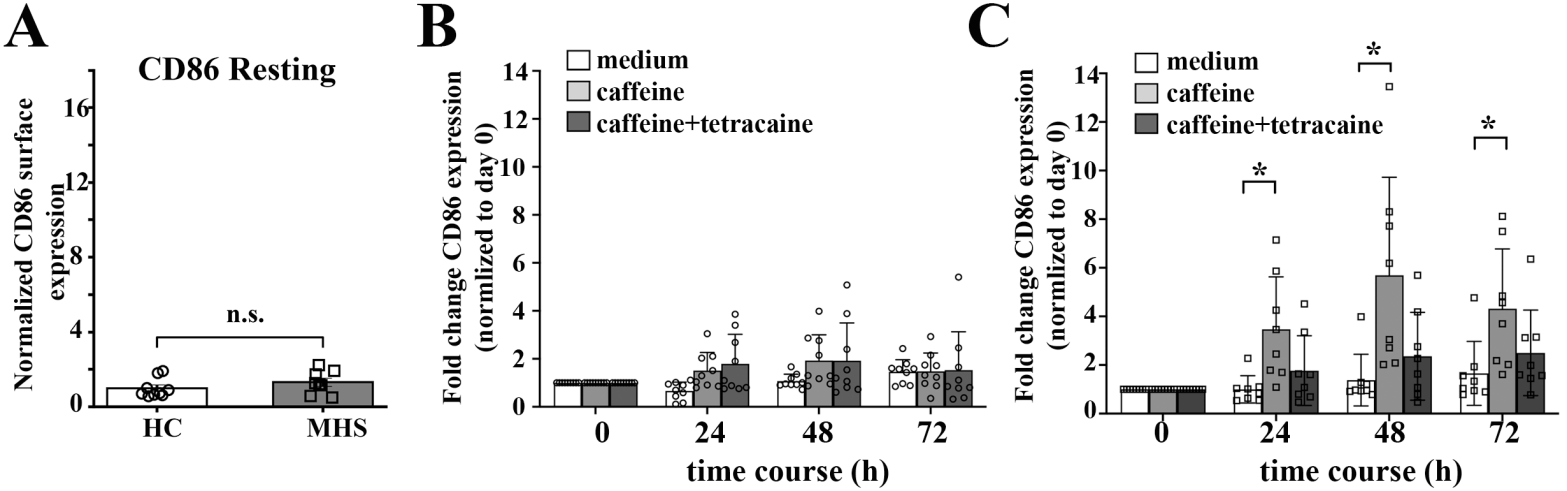
Caffeine induces surface expression of CD86 in EBV immortalized B cells and this is more pronounced in MHS individuals than HC. **(A)** Bar graph showing the baseline surface expression of CD86, on EBV-immortalized B cells derived from healthy controls (HC, white bars, n = 9) and MHS individuals (grey bars, n= 8). The expression level was normalized to the Mean Fluorescence Intensity (MFI) of unstained cells. **(B, C)** Bar graphs showing the time-dependent fold change in CD86 surface expression (at t=0, t=24, t= 48, and t=72 hours) in EBV immortalized B cells from HC (n=9 cell lines) [panel **(B)**] and MHS (n=8 cell lines) [panel **(C)**]. White bars, cells incubated in medium alone; light grey bars, cells incubated in medium containing 10 mM caffeine; dark grey bars cells incubated in medium containing 10 mM caffeine plus 150 µM tetracaine. The bars shown in panels **(B, C)** are the mean (± s.d.) of 3 independent experiments. The CD86 surface expression levels presented in panels **(B, C)** were normalized to the MFI observed at t=0. Statistical comparisons between HC and MHS groups for resting data [panel **(A)**] were performed using the Mann-Whitney U test. Statistical comparisons evaluating the effects of treatment conditions over time in individual cell lines [panels **(B, C)**] were performed using a two-way ANOVA followed by the Bonferroni *Post hoc* test. *p< 0.05. n.s. means not significant.

In order to demonstrate that the altered calcium homeostasis brought about by the presence of *RYR1* mutations is causally involved in the increased surface expression of CD86 observed in the PBMCs, we designed experiments using EBV-immortalized B-cells. EBV primarily infects CD19^+^CD27^+^ B memory subsets (27), inducing them to continuously proliferate but in doing so may cause changes in surface markers expression. Though the results depicted in **Figure 3** demonstrate that circulating B cells from MHS individuals exhibit higher surface expression levels of CD86 and CD40, in EBV-immortalized B-cells the levels of all surface markers investigated, including CD86, were similar between MHS (grey bar, n=8) and HC (white bar, n=9) (**Figure 6**). We stimulated the cells with the RyR1 agonist 4-cmc but this resulted in cell toxicity after several hours. We therefore stimulated the cells with another commonly used RyR1 agonist, namely caffeine (28). The latter agonist was used in multiple studies demonstrating its ability to stimulate RyR1-mediated calcium release from EBV-immortalized B cells and human dendritic cells (19, 29). EBV-immortalized cells from HC and MHS individuals were incubated in the presence of 10 mM caffeine, a concentration at which EBV-immortalized B cells from healthy controls and patients carrying *RYR1* mutations have been previously shown to exhibit max calcium release (23, 24, 29). Cells were harvested at times 0, 24, 48 and 72 hours post stimulation and examined for surface marker expression. Caffeine did not induce any significant time-dependent changes in CD83 surface expression (**Supplementary Figure 7A**) or CD40 (**Supplementary Figure 7B**), however, there was a caffeine-induced time dependent increase in surface expression of CD86, which peaked at 48 hours and subsequently declined. The effect was more pronounced in cells from the MHS cohort (light grey bars, **Figure 6**) than in the HC (light grey bars, **Figure 6**) at all time points (at 24 h CD86 surface expression was 3.47 ± 2.16 vs 1.51 ± 0.75 in MHS vs HC, p=0.021; at 48 h CD86 expression was 5.69 ± 4.03 vs 1.93 ± 1.08, p= 0.021 in MHS vs HC; at 72 h CD86 surface expression was 4.32 ± 2.46 vs 1.48 ± 0.76 in MHS vs HC, Mann-Witney U test p=0.004).

Incubation of cells in the presence of 10 mM caffeine plus 150 µM of the RyR1 inhibitor tetracaine (17, 28) abolished the caffeine-induced CD86 surface expression (**Figures 6** dark grey bars). In these experiments we could not use dantrolene as the RyR1 inhibitor of choice since its presence strongly interferes with fluorescence measurements, making us select tetracaine to inhibit RyR1. Xestospongin C (2 µM), an inhibitor of the InsP3 receptor-mediated calcium release (30) had no effect on caffeine-induced CD86 expression in EBV- cells from HC (**Supplementary Figure 7C**) or from MHS (**Supplementary Figure 7D**) (see Supplementary Supporting data for all mean ± s.d and exact p values).

In conclusion, the results obtained in this section confirm that there is a causal relationship between surface expression of CD86 on B-lymphocytes and RyR1 dependent calcium release. The results also confirm that stimulation of cells from MHS individuals carrying *RYR1* mutations results in significantly higher levels of CD86 surface expression than cells from HC. **Supplementary Table 4** combines the genetic status, allergy predisposition and immune markers at the individual level providing an overview of the potential effects of specific mutations on the immune system.

## 3 Discussion

In the present study we explored whether the MHS phenotype is accompanied by changes in the immune system, by investigating a large cohort of Brazilian Malignant Hyperthermia Susceptible individuals and healthy controls. Compared to recently published data on the frequency of allergies in the Brazilian population, Brazilian MHS individuals have a trend towards more frequent allergic rhinitis. They also have altered cytokine levels when compared to a healthy control cohort which included non-affected (MHN) family members and unrelated healthy controls. Additionally, circulating B-cells from the MHS cohort express higher surface levels of the co-stimulatory molecules CD86 and CD40. These results support the hypothesis that the presence of mutant, hypersensitive RyR1 calcium release channels in immune cells, particularly in antigen-presenting B-cells is associated with a pre-activated immune system, better equipped to mount an immune response during the early stages of infection, but which can also predispose to allergy development.

To date only a few studies have investigated the impact of *RYR1* mutations on the immune system, for example: (i) dendritic cells isolated from Y522S *Ryr1* knock in mice were phenotypically more mature, more efficient at stimulating T cell proliferation, and were more efficient at mounting a specific immune response in the early phases of immunization compared to those from WT littermates (21). (ii) Mice knocked in for the p.R163C *Ryr1* mutation were more susceptible to experimentally induced autoimmune encephalomyelitis than their WT littermates, and administration of the dantrolene dampened the autoimmune reaction (20). (iii) Pigs with the naturally occurring homozygous p.R614C mutation developed a greater level of immune defense and greater NK cytotoxic activity than heterozygous or non-mutation carrying pigs (31). These studies on animal models strongly support the hypothesis that *RYR1* mutations influence immune functions, but have the shortcoming of being carried out on inbred animal models kept under germ-free conditions or a protected laboratory environment, and thus may not match what occurs in a genetically diverse human populations continuously exposed to antigenic challenges. The fact that we were able to observe differences between the frequency of individuals with some allergies as well our preliminary observation of reduced rates of influenza and COVID-19 infections in the genetically diverse Brazilian MHS cohort, substantiate the hypothesis that RyR1 mutations play a role in immunity. Nevertheless, these results must be interpreted with caution as they are based on a questionnaire study that has the limitation of being established on self-reported allergies and comparisons across non-identical cohorts (our Brazilian cohort versus the published Brazilian cohort).

RyR1 has been identified in human immune cells including B-lymphocytes, T-lymphocytes, monocytes (32), plasmacytoid cells and *in vitro*-derived dendritic cells (19). In CD3^+^ T-lymphocytes, generation of the second messenger NAADP leads to the activation of RyR1-mediated Ca^2+^ release generating a positive feedback mechanism to boost T cell receptor-induced Ca^2+^ signaling (33), and in mice experimentally-induced arthritis was significantly reduced by pretreatment with the RyR1 antagonist dantrolene prior to bovine collagen II injections (34). Interestingly, T cells from p.R163C *Ryr1* knock in mice display enhanced calcium leakage from intracellular stores (35), though how the p.R163C RyR1 mutation impacts T-cell function or the immune response has not been investigated. We would like to point out that this is the first report showing that immune functions of human MHS individuals differ from those of healthy controls. A limitation of the results from the allergy questionnaire is that there was no significant difference between the MHS and MHN family members/healthy control group. This could be due to the small sample number of HC cohort (n=31) and to the fact that 71% of the HC cohort were females, whereby the risk of developing allergies and autoimmune diseases is far greater in adult females than males pointing to a potential role of sex hormones in the development of atopic disorders (36). For these reasons, we expanded the HC cohort using previously published data in the same Brazilian population as the MHS cohort and with the same ratio of males and females (22). Serum samples from MHS individuals showed altered levels of cytokines, lower levels of IgM but we were unable to demonstrate higher levels of IgE, as was observed in the Y522S *Ryr1* knock-in mice (21). The lack of measurable IgE in the human serum samples is most likely due to the fact that it is an extremely labile immunoglobulin, having a half-life of 2–3 days (37) and its serum levels in all the Brazilian subjects may be low because of the time required to collect blood samples in patients living all over Brazil and shipping them to the MH lab in São Paulo and then to the Clinical Laboratory of the University Hospital in Basel.

Cytokines are key soluble regulators of the immune system, and their presence is often indicative of an altered immunological state. For example, the presence of high circulating levels of IL-1 and IL-6 are indicative of inflammation (38), IL-4 is a marker of enhanced type 2 reactions, of allergies and chronic inflammation (39). IL-10 on the other hand, has predominantly anti-inflammatory activities inhibiting allergic reactions (40) and its higher levels in the serum of the MHS cohort may indicate the activation of a feedback inhibitory mechanism to lessen an over-reactive immune response. In the context of IL-10, there is an apparent contradictory finding between the elevated concentrations of this cytokine in the serum of the MHS cohort compared to HC, but whose secretion by EBV-immortalized B cells was lower in cells from the MHS cohort. Infection of B-cells with EBV, leads to production of viral IL-10 (41) which is antigenically similar to human IL-10, however the viral load of the MHS and HC cell lines was similar, excluding this as the reason for the lower IL-10 secretion levels by MHS EBV-immortalized cell lines. The contradictory results relating to serum IL-10 and IL-10 released by MHS-derived EBV cell lines may reflect the fact that this cytokine is produced by a large array of immune cells commonly associated with chronic immune activation (42, 43) and that EBV primarily infects CD19^+^CD27^+^ memory B cells (27). Under physiological conditions memory B-cells are predominantly located in the spleen, lymph nodes and other secondary lymphoid organs where they become rapidly activated following exposure to a previously encountered antigen. Under normal physiological conditions memory B cells would release cytokines in a closed microenvironment where the secondary immune response is activated and where memory B cells can become Ig secreting plasma cells. In the microenvironment of the secondary lymphoid organs the low IL-10 release by B-cells could favor the development of an allergic immune phenotype, since IL-10 producing B cells normally suppress allergic responses (44). Thus, decreased levels of IL-10 released by memory B-cells in the cell lines derived from MHS-individuals are compatible with reduced immune tolerance and a shift to a less tolerogenic phenotype.

In the present investigation the number of serum donors from the MHS cohort was small so that no definitive correlation can be conclusively drawn between the MHS phenotype and high levels of circulating cytokines. Nevertheless, in a previous study we showed that individuals with MHS carrying *RYR1* mutations had significantly higher circulating levels of IL-6 compared to healthy controls (45) substantiating the present results. IL-6 can be produced by various cell types, including monocytes and macrophages, adipocytes and skeletal muscle (46–48). Skeletal muscles release IL-6 following exercise and cultured myotubes release IL-6 following pharmacological RyR1 activation (48, 49). Though at present this remains a speculation, it is possible that the high IL-6 levels are causally linked to the development of anesthesia-induced Malignant Hyperthermia. In fact, the probability of developing an MH reaction in *RYR1*-mutation carrying individuals was reported to be significantly higher in subjects who did intense exercise or reported a viral illness 72 hours prior to the triggering anesthesia than in *RYR1*-mutation carrying subjects who did not trigger (50) and both exercise and viral infections would result in elevated circulating levels of IL-6. At this point in time however, no firm conclusions can be drawn regarding the higher circulating levels of IL-4, IL-10 and IL-6 and future studies investigating a larger number of MHS subjects may help clarify this point.

In murine B-lymphocytes surface expression of CD86 could be induced either by incubation with anti-IgM (mimicking B cell receptor activation) or incubation with ionomycin, indicating that it is a calcium-dependent event (51). Here we show that circulating B cells from the MHS cohort exhibit a higher surface expression of CD86 (and CD40) than HC and that in EBV-immortalized B cells, membrane expression of CD86 can be induced in a time-dependent manner by incubation with caffeine. CD86 surface expression is sensitive to the RyR1 blocker tetracaine but is unaffected by the InsP3R antagonist Xestospongin C. The apparent discrepancy between the elevated surface expression of CD86 in circulating B cells and the unchanged surface expression of CD86 in resting EBV-immortalized B-cell lines may be due to several factors, including *in vitro* adaptation of the cell cultures, continuous stimulation of CD86 surface expression induced by EBV, the fact that EBV-immortalized B cell lines arise mostly from memory B cells (27) which typically express higher resting levels of CD86 (52), or a combination of these factors.

Though we do not know what the physiological activator of the RyR1 in B-cells is, it is plausible that it is the Ca^2+^ influx mediated by STIM1-Orai1 activation following B cell receptor engagement that activates the RyR1 via the mechanism of Ca^2+^-induced Ca^2+^-release, resulting in down-stream events leading to higher surface expression of CD86. The results of previous studies showing that *RYR1* mutations increase CD83 surface expression on dendritic cells (21), as well as those of the present investigation also suggest an interesting mechanism by which *RYR1* mutations may lead to the predisposition to develop allergies. CD86 is a crucial molecule involved in the activation of CD4^+^T_H_ cells by antigen-presenting cells, whereby CD86 interacts with CD28 to trigger T cell activation, proliferation and IL-2 production during the early phases of the immune response (53, 54). CD86 works in tandem with CD40, whereby the binding of CD40 to CD40L/CD154 on activated CD4^+^T_H_ cells provides further co-stimulatory signals to T cells, and also leads to increased CD86 expression on antigen-presenting (B)-cells (55). It is possible that higher surface expression levels of CD86, CD40 and CD83 results in a more efficient signal that activates CD4^+^T_H_ cells even when the interaction of the T cell receptor with the MHC class II molecules peptide would be insufficient to generate a strong activating signal in CD4^+^T_H_ cells. In line, silencing CD40 and CD86 on dendritic cells was shown to have an inhibitory effect of allergic responses in mice, reducing sneezing and nasal rubbing (56).

We are aware that the size of the population investigated in the present study is small and that the number of individuals enrolling in all aspects of the study does not allow us to draw firm conclusions. Nevertheless, this is the first study on the characterization of the human immune phenotype associated with MHS-linked *RYR1* variants and together with previous studies on transgenic mouse models knocked in for MHS-linked *RYR1*-mutations, we provide evidence for a link between *RYR1* mutations and alterations of the immune system. The predisposing factors to developing allergies are multifaceted, involving sex, environmental factors (such as exposure to pollutants, pollens, dust mites, etc) and genetics. Understanding these factors will help identify individuals at risk, mitigate the onset of allergic disease and help develop new classes of drugs. Many genes have been implicated in the predisposition to develop allergies including genes encoding proteins of the skin barrier, immune response, antigen presentation, airway hyperreactivity, and the results of the present study suggest that *RYR1* mutations, which occur with a frequency of approximately 1:3000 individuals, should also be considered as a potential (co-)cause of allergic predisposition, because the mutations subtly alter calcium homeostasis in immune cells. The increase of CD86 and CD40 drives enhanced antigen presentation and T-cell co-stimulatory capacity.

These events may have been an evolutionary advantage for carriers of gain of function *RYR1* mutation in the pre-antibiotic era. The findings of the present investigation also raise the intriguing possibility of adding RyR1 activators in order to enhance the immunogenicity of vaccines in immunocompromised individuals.

## 4 Materials and methods

### 4.1 Patient selection

We reviewed the data from all families referred for evaluation in the Brazilian MH unit between 1997 and 2025, in order to invite those with a personal/family history of MH linked to anesthesia. Demographic and clinical data were collected from 2021 to 2025, as well as the results of the *in vitro contracture test* (IVCT) according to the EMHG protocol (13).

### 4.2 Sex as a biological variable

sex was not considered as a biological variable and both male and female individuals were equally included in this study. In the context of the allergy questionnaire, 51.4% of the enrolled MHS individuals were females whereas females represented 71% of the HC individuals. This unbalance between the percentage of females and males, motivated us to compare the data of the MHS group to published data on autoimmune disorders in the Brazilian population.

### 4.3 Allergy questionnaire

Enrolled patients completed the immune system questionnaire, including the allergy specific survey, influenza and COVID-19 viral infections query, and adverse reactions to influenza and COVID-19 vaccination. The allergy questionnaire was adapted from a Brazilian instrument for allergy screening (57). The questionnaire was auto-applicable, delivered online for participants at home. Study data were collected and managed using REDCap electronic data capture tools hosted at the University Federal of São Paulo. REDCap (Research Electronic Data Capture) is a secure, web-based software platform designed to support data capture for research studies, providing 1) an intuitive interface for validated data capture; 2) audit trails for tracking data manipulation and export procedures; 3) automated export procedures for seamless data downloads to common statistical packages; and 4) procedures for data integration and interoperability with external sources (58, 59).

### 4.4 PBMC isolation and cryopreservation

PBMCs were isolated by density-gradient centrifugation using Ficoll-Paque (Sigma-Aldrich Catalog N° GE17-1440-02). Briefly, blood was diluted 1:1 with phosphate buffered saline (PBS, ThermoFisher Scientific Catalog N°10010023), PBMC were collected, washed twice (300 ×g, 10 min) in PBS or RPMI 1640 (Sigma-Aldrich Catalog N° R8758), and cells were counted. Fresh PBMCs were either used immediately for immunophenotyping or resuspended at 5–10×10^6^ cells/ml in fetal bovine serum (FBS, ThermoFisher Scientific Gibco™, Catalog N° A5256701) containing 10% dimethylsulphoxide (DMSO, Sigma-Aldrich Catalog N° 41639) and gradually frozen in a controlled-rate freezing container at −80 °C before transfer to liquid nitrogen for long-term storage.

### 4.5 Generation and culture of EBV-immortalized B cell lines

PBMCs were obtained from MHS patients and healthy controls (HCs) from two cohorts. The Brazilian cohort included individuals positive for the *in vitro* contracture test (IVCT) carrying the MHS-associated RyR1 variants listed in **Supplementary Table 3**; one IVCT-positive donor with a yet to be identified *RYR1* variant. EBV-immortalized B cell lines were generated from healthy Brazilian donors (healthy controls, HC) who were either family members of the MHS individuals that did not carry any *RYR1* mutations, or other unrelated HC. To expand the cohort of MHS individuals, we also incorporated a Swedish MHS cohort carrying previously characterized RyR1 variants as well as a Swedish HC (26). EBV-immortalized B-lymphocytes were obtained by infecting PBMCs with a supernatant derived from B95–8 marmoset lymphoblastoid cells as previously described (24, 26). Cultures were maintained in growth medium consisting of RPMI-1640 (Sigma Aldrich, catalog N° R8758) containing 10% FBS ThermoFisher Scientific Gibco™, Catalog N°A5256701), 1% Glutamax (ThermoFisher Scientific, Catalog N° 35050038), 1% (1g) Glucose (ThermoFisher Scientific, Catalog N° A2494001), 1mM sodium pyruvate (ThermoFisher Scientific Catalog N° 11360039), 2mM HEPES (ThermoFisher Scientific Catalog N° 15630056) and 1% penicillin–streptomycin (ThermoFisher Scientific Catalog N° 15140122) for 3 weeks, with weekly medium changes and cyclosporine supplementation (1 μg/mL, Merk catalog N° 30024–25 mg), followed by cyclosporine withdrawal once stable proliferating B-lymphocytes cell lines were established. Mature lines were frozen in CryoStor and stored in liquid nitrogen for long-term preservation. For functional assays, all EBV-immortalized B-lymphocyte cell lines were maintained in logarithmic growth phase and used at low-passage numbers to minimize experimental variability. Cell identity and EBV transformation efficiency were verified by immunophenotyping (CD19^+^/CD20^+^, EBNA-1^+^).

### 4.6 Quantification of serum immunoglobulin levels, cytokines and CMV avidity assessment

Serum immunoglobulin levels were measured using a bead-based immunoassay combined with flow cytometry by the clinical laboratory of the Basel University Hospital according to standardized diagnostic protocols. Data are plotted as mean (± s.d.). Because IgA subclasses could not be distinguished by the bead-based assay, serum IgA2 levels were measured separately using an enzyme-linked immunosorbent assay (Abcam, Catolog N° ab315787) according to the manufacturer’s instructions. Absorbance was measured using a microplate reader, and IgA2 concentrations were calculated from a standard curve generated using purified IgA2 standards.

CMV IgG avidity testing was performed by the Clinical Laboratory of the University Hospital on previously collected patient serum samples. The assay was conducted using a commercial chemiluminescent immunoassay (CLIA) (LIAISON^®^ CMV IgG Avidity II, DiaSorin, Italy) according to the manufacturer’s instructions. Data are plotted as mean (± s.d.).

Cytokines were quantified by ELISA using commercially available kits (**Supplementary Table 5**), following the manufacturer’s recommendations. Each quantification was performed in duplicate and the cytokine concentration was determined using a standard curve generated using cytokine standards included in the kits. Results are plotted as mean (± s.d.).

### 4.7 Flow cytometric immunophenotyping of PBMCs

Cryopreserved peripheral blood mononuclear cells (PBMCs) were washed with ice-cold PBS immediately after thawing and then directly stained on ice with a multicolor antibody panel (**Supplementary Table 6**) for identification of major immune cell populations and B-cell subpopulations. All staining procedures were done on ice to reduce unspecific activation of cells and internalization of surface markers. A viability dye (ThermoFisher Scientific, LIVE/DEAD™ Fixable Blue, Catalog N° L23105) was included in all samples to exclude dead cells before analysis.

### 4.8 Gating and identification of major immune cell populations

Flow cytometry data were acquired on a multicolor flow cytometer (Cytek Aurora) and analyzed using FlowJo software. Spectral unmixing was performed on the Cytek SpectroFlo^®^ software using UltraComp eBeads™ Compensation Beads (Thermo Fisher, Catalog N° 01-2222-42). Lymphocytes were first gated based on forward scatter area (FSC-A) and side scatter area (SSC-A). Doublets were excluded using FSC-A versus FSC-H gating, followed by exclusion of dead cells using the viability dye. Live singlet cells were then re-gated on FSC-A versus SSC-A to define the total cell population used for further analysis. Major immune cell populations were identified using standard surface lineage markers. T cells were defined as CD3^+^ cells and B cells were defined as CD19^+^ cells. NK cells were identified as CD3^−^CD19^-^CD56^+^ cells, with additional separation based on CD56 expression when applicable. Myeloid cells were defined as CD56^-^HLA-DR^high^CD14^+^ cells. The remaining CD3^−^CD19^−^CD14^−^ cells were classified as other cells and were not included in lineage-specific analysis (see **Supplementary Figure 1A**).

### 4.9 B-cell identification and subset definition

Total B cells were gated as CD19^+^ cells within the live singlet lymphocyte population. CD19 low and CD20^−^ events were gated separately and excluded from conventional B-cell subset analysis. Conventional B cells were defined as CD19^+^CD20^+^ cells and further subdivided based on CD27 and IgD expression. Naïve B cells were defined as CD27^−^IgD^+^CD10^−^ cells. Memory B cells were defined as CD27^+^ cells and further divided into unswitched memory (CD27^+^IgD^+^) and switched memory (CD27^+^IgD^−^) B cells. Transitional B cells were identified CD27^−^IgD^+^CD10^+^. Effector B-cell populations were identified as CD38^-^CD21^-^(**Supplementary Figure 1B**).

### 4.10 Activation and functional marker analysis

Expression of activation and functional markers, including CD86, CD40, CD83, PD-L1, BAFF-R, CD71, CD85j, HLA-DR, CD39, IgM, IgA and IgG were analyzed on total B cells and defined B-cell subsets. Because flow cytometry experiments were performed in two separate runs, the median fluorescence intensity (MFI) of each sample was normalized to the no-staining negative samples from the same run to reduce batch-related variation. The normalized values were used for downstream analysis. For statistical comparison of normalized MFI values between HC and MHS groups, multiple B-cell subset markers were analyzed simultaneously, therefore statistical analysis was performed using the Benjamini–Hochberg False Discovery Rate (FDR). For **Figure 3B**, correction was performed with a setting of m=6, corresponding to the 6 B-cell subsets that were compared simultaneously between HC and MHS groups. For **Figures 3C, D**, correction was also performed with a setting of m = 7, corresponding to the whole B-cell population and 6 B-cell subsets tested simultaneously, including activation markers (CD86, CD40, CD83, MHCII, CD71 and BAFFR), regulation markers (PD-L1, CD39 and CD85j) and surface immunoglobulins (IgM, IgD, IgG and IgA). While CD86 and CD40 results are presented in the main figures (**Figures 3C, D**), comparisons for the remaining markers are provided in **Supplementary Figure 2**.

The Benjamini–Hochberg procedure was implemented by ranking raw p-values in ascending order (p_1_ ≤ p_2_ ≤ … ≤ p_m_) and calculating adjusted values according to:


$$qi=\frac{\left(pi\times m\right)}{i}$$

where *i* represents the rank of the individual test and m represents the total number of comparisons within the respective panel (m=6 or m=7). Monotonicity correction was applied to ensure non-decreasing adjusted values across ranks. Adjusted p-values are reported as q-values. Statistical significance for multiple-testing panels was defined as q< 0.05.

### 4.11 Analysis of surface markers on EBV-immortalized B cells

EBV-immortalized B cell lines derived from MHS patients and HC were seeded at a density of 1.0×10^6^ cells/ml in complete RPMI. Cells were stimulated with 10 mM caffeine (Sigma-Aldrich, Catalog N°C0750) to activate RyR1-dependent Ca^2+^ release. Where indicated, the following pharmacological inhibitors were used: 150 µM tetracaine (ThermoFisher Scientific, Catalog N° 460821000) to inhibit RyR1 (17, 28) and 2 µM Xestospongin C (Abcam, Catalog N° ab120914) to inhibit InsP3-dependent Ca^2+^ release (30). Untreated cells served as baseline controls. After stimulation, cells were placed back in the cell culture incubator (5% CO_2_, 37 °C) for 24, 48, and 72 h, harvested, washed with PBS (ThermoFisher Scientific, Catalog N° 20012019), and stained on ice with fluorochrome-conjugated monoclonal antibodies directed against the B-cell activation marker CD86, CD83 and CD40 (**Supplementary Table 6**). Cell viability was assessed and determined to be >90%. After staining and washes, cells were fixed with 4% PFA in PBS and measured on a multi-laser flow cytometer (Cytek Aurora). Mean fluorescence intensity (MFI) values were extracted for gated single B-cells. For baseline resting surface marker expression, the Mean Fluorescence Intensity (MFI) of each marker was normalized to the MFI of unstained control cells to account for autofluorescence; for post-stimulation surface marker expression, the MFI was normalized to the MFI at t=0 h for each cell line. Kinetic curves were generated for each condition, and differences in surface marker expression between MHS and HC lines, and between drug treatments, were evaluated over time. Experiments were performed in triplicate and are plotted as mean fluorescent intensity (± s.d.).

### 4.12 Cytokine release from EBV-immortalized B lymphocytes

1.5x10^6^ cells from each cell line were washed with PBS and centrifuged at 300g for 7 minutes. Cells were then resuspended in fresh RPMI 1640, seeded in 6-well tissue culture dishes and incubated at 37 °C for 16 hours. The cells and their supernatants were collected in 1.5 ml Eppendorf tubes, filtered through low protein binding, PVDF 0.45 µm filters (MilliporeSigma Millex-HV, SLHVR33RS) to ensure cell removal, flash frozen and stored at -80 °C. Cytokine quantification was carried out by sandwich ELISA as described below. The day before the experiment, 96-well microplates (ThermoFisher Scientific, Catalog N° 442404) were coated with 50 µl per well of capture antibodies (**Supplementary Table 5**) diluted 1:100 in filtered PBS and incubated overnight at 4 °C. The next day, the capture antibody was removed, the plates were washed three times with 300 µl per well PBS-T and any remaining wash buffer was removed. The wells of the plates were blocked with 100µl 1%SureBlock blocking buffer (LubioScience, Catalog N° SB232010) in PBS for 90 minutes at room temperature. The blocking buffer was then removed, and the wells were washed 3 times with PBS-T. Samples to analyze were thawed on ice and diluted 1:2 with 1% SureBlock solution. The following recombinant human cytokine standards were used (BioLegend) and standard curves in the range of 4 to 500 pg/ml were generated: IL-2 (Catalog N° 79040), IL-4 (Catalog N° 79022), IL-6 (Catalog N° 79028), IL-10 (Catalog N° 79031), IL-12 p70 (Catalog N° 79037), IL-1α (Catalog N° 78155), IL-1β (Catalog N° 78164), and TNF-α (Catalog N° 79019). One hundred microliters of each sample were added to the ELISA wells and incubated at room temperature for 2 hours. The samples were then removed and the plates were washed with 300 µl PBS-T 3 times as described above. Secondary enzyme coupled detection antibody (**Supplementary Table 4**) were prepared by diluting them 1:250 in 1% SureBlock solution, and 100 µl of the diluted antibodies were added to each well and incubated at room temperature in the dark for 1 hour. The wells were washed three times with PBS-T, and 100 µl of 1:2000 diluted HRP-streptavidin (BioLegend, Catalog N° 405210) in 1% SureBlock solution was added into each well. The plate was incubated for 1 hour in the dark, followed by 3 washes with 300 µl PBS-T. Finally, 50 µl of the 1-Step™ Ultra TMB-ELISA Substrate Solution (Thermo Fisher Scientific, Catalog N° 34028) equilibrated at room temperature were added to each well. After 20 min, 50 µl of stop solution (2M Sulfuric Acid) were added to each well and plates were read in a plate reader (BioTek, Synergy H1 Hybrid Reader) at an absorbance of 450 nm. The average cytokine concentration in each sample (experimental duplicates) was calculated using the linear equation derived from the standard curves and the results are expressed as mean (± s.d.) cytokine concentration.

### 4.13 Quantification of Epstein Barr viral load in each cell line

2 x10^6^ cells were harvested by centrifugation (300x g for 7 minutes), washed once with PBS, and the cell pellets were used for total RNA isolation following the protocol provided in the Direct-zol™ RNA MicroPrep kit (Zymo Research, Irvine, CA, USA Catalog N° R2062). Concentration and purity of isolated RNA were assessed using a NanoDrop Spectrophotometer. Total RNA (1 µg) was reverse transcribed into cDNA using, High-Capacity cDNA Reverse Transcription Kit (ThermoFisher Scientific Catalog N° 4368814) using an Applied Biosystems 2720 Thermal Cycler (ThermoFisher Scientific). To evaluate EBV viral load across all immortalized cell lines, qPCR was performed targeting the viral gene *EBNA1*. Reactions were prepared using PowerUp™ SYBR™ Green Master Mix (ThermoFisher Scientific Catalog N° A25742) with 10 ng of cDNA using an Applied Biosystems™ 7500 Fast Real-Time PCR System (ThermoFisher Scientific). The thermal cycling conditions were set as follows: initial hot start (95 °C for 2 minutes), followed by 40 cycles of denaturation at 95 °C for 15 seconds and an annealing/extension step at 60 °C for 1 minute. Expression levels were normalized to the housekeeping gene *GAPDH*. The following primer sequences were used: *EBNA1*: Forward 5’-TCATCATCATCCGGGTCTCC-3’ and Reverse 5’-CCTACAGGGTGGAAAAATGGC-3’ *GAPDH*: Forward 5’-GAGAAGGCTGGGGCTCATTTT-3’ and Reverse 5’-ATGACGAACATGGGGGCATC-3’. Each symbol represents the mean (± s.d.) of three separate experiments. *EBNA1* expression of HC was set as 1.

### 4.14 Cell proliferation

Cell proliferation was assessed using the CellTiter 96^®^ AQueous One Solution Cell Proliferation Assay (MTS) (Promega, Catalog N° G3582). To ensure optimal signal detection within the linear range, a seeding density of 5x10^4^ cells per well was used. Technical duplicates were plated in 96-well plates and incubated for 0 h (T_0_) and 48 h (T_48_). Following the addition of the MTS reagent, the absorbance was measured at 490 nm using a Synergy H1 Hybrid Multi-Mode Microplate Reader (BioTek Instruments, Winooski, VT, USA). The proliferation index was calculated as the ratio of the optical density (OD) at 48 h to the OD at 0 h (OD T_48/_OD T_0_). Each symbol represents the mean (± s.d.) of three separate experiments.

### 4.15 Intracellular Ca^2+^ measurements and EC_50_ determination

Ca^2+^ release via RyR1 activation was quantified in EBV-immortalized B cells, with the fluorescent indicator Fluo-4 (Invitrogen Catalog N°F14201). Briefly, 1x10^6^ cells/ml cells were loaded with Fluo-4 AM (5 µM) in Hanks Balanced Salt Solution (HBSS, ThermoFisher Scientific Catalog N° 14175) for 30–45 min at 37 °C, followed by a de-esterification step in HBSS for 10 min at 37 °C. Baseline fluorescence was recorded for each cell line before the addition of the specified concentration (100–950 µM) of the RyR1 activator 4-chloro-m-cresol (4-cmc, Sigma Aldrich, Catalog N° 24940). Fluorescence was monitored in real time using a plate reader (BioTek Synergy H1) (excitation 488 nm, emission 515–530 nm). Relative fluorescence units (RFU) were normalized to baseline (ΔF/F_0_), and the peak responses to each 4-cmc concentration were calculated. Dose–response curves were generated by plotting normalized calcium release calculated as % of maximal calcium release, induced by 900 µM 4-cmc versus [4-cmc]. Non-linear regression was performed using a four-parameter logistic model to obtain half-maximal effective concentration (EC_50_) values for each cell line. EC_50_ distributions were compared between HC and MHS groups to assess the sensitivity of the 4-cmc-induced Ca^2+^ release.

### 4.16 Statistical analysis

All statistical analyses were performed using GraphPad Prism and/or Python. Data are presented as mean (± s.d.) unless otherwise indicated. For comparisons between two independent groups (e.g. HC vs MHS cell lines or donors), unpaired t tests or non-parametric Mann–Whitney U tests were used. To control for false positive due to multiple simultaneous comparisons within defined experimental panels (e.g., flow cytometry multi-marker immunophenotyping of PBMC), raw p-values were adjusted using the Benjamini–Hochberg False Discovery Rate (FDR). The number of comparisons (m) was pre-specified as the total number of biologically interrogated variables within each experimental panel. Markers used solely for population gating were not included in the multiple-testing correction. Adjusted p-values are reported as q-values. For kinetic experiments (e.g. CD86 expression over time with caffeine ± inhibitors), repeated-measurements two-way ANOVA tests were used. For EC_50_ analyses, logistic curve fits were generated for each cell line, and EC_50_ values were compared between MHN and MHS groups using unpaired tests. Statistical significance was defined as p<0.05 for single comparisons and q<0.05 for analyses involving multiple testing. Exact statistical tests, n values, and significance levels are reported in the text. Allergy comparisons, including the prevalence of overall allergies and specific allergy types (e.g., rhinitis), were analyzed using Fisher’s Exact test followed by the FDR corrected p value.

### 4.17 Study approval

The study received the Federal University of São Paulo Research Ethics Committee approval (N° 0682P/2021). Patients gave informed consent for anonymized publication of their clinical information prior to participation in the study.

### 4.18 Equations


$$\text{qi}=\frac{\left(\text{pi}\times \text{m}\right)}{\text{i}}$$

In the equation *m* corresponds to cell subsets. In figure 3B, *m* = 6 corresponding to the six B-cell subsets compared simultaneously between the HC and MHS groups, namely: naïve, transitional, non-switched memory, switched memory, effector and regulatory. For **Figures 3C, D**, correction was applied with *m* = 7, corresponding to the whole B-cell population plus six B-cell subsets tested simultaneously, namely: whole B cells, transitional B cells, naïve B cells, non-switched memory B cells, switched memory B cells, effector B cells, and regulatory B cells (Breg).

## Data Availability

The original contributions presented in the study are included in the article/**Supplementary Material**. Further inquiries can be directed to the corresponding author.

## References

[1] Berridge MJ Lipp P Bootman MD . Calcium signalling: dynamics, homeostasis and remodelling. Nat Rev Mol Cell Biol. (2000) 1:11–21. doi: 10.1038/nrm1155 12838335

[2] Oh-hora M Rao A . Calcium signalling in lymphocytes. Curr Opin Immunol. (2008) 20:250–8. doi: 10.1016/j.coi.2008.04.004 18515054 PMC2574011

[3] Lewis RS . Calcium signaling mechanisms in T lymphocytes. Ann Rev Immunol. (2001) 19:497–521. doi: 10.1146/annurev.immunol.19.1.497 11244045

[4] Baba Y Kurosaki T . Role of calcium signaling in B cell activation and biology. Curr Topics Microbiol Immunol. (2016) 393:143–74. doi: 10.1007/82_2015_477 26369772

[5] Vaeth M Kahlfuss S Feske S . CRAC channels and calcium influx in T cell mediated immunity. Trends Immunol. (2020) 41:878–901. doi: 10.1016/j.it.2020.06.012 32711944 PMC7985820

[6] Vig M Kinet JP . Calcium signaling in immune cells. Nat Immunol. (2009) 10:21–7. doi: 10.1038/ni.f.220 19088738 PMC2877033

[7] Park YJ Yoo SA Kim M . The role of calcium-calcineurin-NFAT signaling pathway in health and autoimmune diseases. Front Immunol. (2020) 11:2020. doi: 10.3389/fimmu.2020.00195 32210952 PMC7075805

[8] Meissner G . Ryanodine receptor/Ca2+ release channels and their regulation by endogenous effectors. Ann Rev Physiol. (1994) 56:485–508. doi: 10.1146/annurev.ph.56.030194.002413 7516645

[9] Fleischer S Inui M . Biochemistry and biophysics of excitation-contraction coupling. Annu Rev Biophys Biophys Chem. (1989) 18:333–64. doi: 10.1146/annurev.biophys.18.1.333 2660829

[10] Jungbluth H Treves S Zorzato F Sarkozy A Ochala J Sewry C . Congenital myopathies: disorders of excitation–contraction coupling and muscle contraction. Nat Rev Neurol. (2018) 14:151–67. doi: 10.1038/nrneurol.2017.191 29391587

[11] Lawal TA Todd JJ Witherspoon JW Bönnemann CG Dowling JJ Hamilton SL . Ryanodine receptor 1- related disorders: an historical perspective and proposal for a unified nomenclature. Skeletal Muscle. (2020) 10:32. doi: 10.1186/s13395-020-00243-4 33190635 PMC7667763

[12] MacLennan DH Phillips MS . Malignant hyperthermia. Science. (1992) 256:789–94. doi: 10.1126/science.1589759 1589759

[13] Hopkins PM Girard T Dalay S Jenkins B Thacker A Patteril M . Malignant hyperthermia. Anesthesia. (2021) 76:655–64. doi: 10.1016/j.mpaic.2014.03.006 33399225

[14] Rosenberg H Pollock N Schiemann A Bulger T Stowell K . Malignant hyperthermia: a review. Orphanet J Rare Dis. (2015) 10:93. doi: 10.1186/s13023-015-0310-1 26238698 PMC4524368

[15] Gonsalves SG Ng D Johnston JJ Teer JK Stenson PD Cooper DN . Using exome data to identify Malignant hyperthermia susceptibility mutations. Anesthesiology. (2013) 119:1043–53. doi: 10.1097/aln.0b013e3182a8a8e7 24195946 PMC4077354

[16] De Crescenzo V Fogarty KE Lefkowitz JJ Bellve KD Zvaritch E MacLennan DH . Type 1 ryanodine receptor knock in mutation causing central core of skeletal muscle also displays a neuronal phenotype. Proc Natl Acad Sci USA. (2012) 109:610–5. doi: 10.1073/pnas.1115111108 22203976 PMC3258591

[17] Lopez RJ Bryne S Vukcevic M Sekulic-Jablanovic M Xu L Brink M . An RYR1 mutation associated with Malignant hyperthermia is also associated with bleeding abnormalities. Sci Signal. (2016) 9:ra68. doi: 10.1126/scisignal.aad9813 27382027

[18] Hosoi E Nishizaki C Gallagher KL Wyre HW Matsuo Y Sei Y . Expression of the ryanodine receptor isoforms in immune cells. J Immunol. (2001) 167:4887–94. doi: 10.4049/jimmunol.167.9.4887 11673493

[19] Bracci L Vukcevic M Spagnoli G Ducreux S Zorzato F Treves S . Ca2+ signaling through ryanodine receptor 1 enhances maturation and activation of human dendritic cells. J Cell Sci. (2007) 120:2232–40. doi: 10.1242/jcs.017590 17567682

[20] Osipchuk N Soulika AM Fomina AF . Modulation of ryanodine receptors activity alters the course of experimental autoimmune encephalomyelitis in mice. Front Physiol. (2021) 12:770820. doi: 10.3389/fphys.2021.770820 35027891 PMC8751758

[21] Vukcevic M Zorzato F Keck S Tsakiris DA Keiser J Maizels RM . Gain of function in the immune system caused by a ryanodine receptor 1 mutation. J Cell Sci. (2013) 26:3485–92. doi: 10.1242/jcs.130310 23704352 PMC3730249

[22] Seite S Taieb C Strugar TL Lio P Curi T . Prevalence of allergies in Brazil and impact on skin- epidemiological study on a representative sample of Brazilian adults. J Cutaneous Disord Med. (2020) 3:180022.

[23] Lyfenko AD Ducreux S Wang Y Xu L Zorzato F Ferreiro A . Two Central Core Disease (CCD) deletions in the C-terminal region of RYR1 alter muscle excitation-contraction (EC) coupling by distinct mechanisms. Hum Mutat. (2006) 28:61–8. doi: 10.1002/humu.20409 16958053

[24] Levano S Vukcevic M Singer M Mater A Treves S Urwyler A . Increasing the number of diagnostic mutations in Malignant hyperthermia. Hum Mutat. (2008) 30:590–8. doi: 10.1002/humu.20878 19191329

[25] Tegazzin V Scutari E Treves S Zorzato F . Chlorocresol an additive to commercial succinylcholine, induces contracture of human Malignant hyperthermia susceptible muscles via activation of the ryanodine receptor Ca2+ channel. Anesthesiology. (1996) 84:1380–5. doi: 10.1097/00000542-199606000-00014 8669679

[26] Vukcevic M Broman M Islander G Bodelsson M Ranklev-Twetman E Müller CR . Functional properties of RYR1 mutations identified in Swedish patients with Malignant hyperthermia and central core disease. Anesth Analg. (2010) 111:185–90. doi: 10.1213/ane.0b013e3181cbd815 20142353

[27] Babcock GJ Decker LL Freeman RB Thorley-Lawson DA . Epstein-Barr virus-infected resting memory B cells, not proliferating lymphoblasts, accumulate in the peripheral blood of immunosuppressed patients. J Exp Med. (1999) 190:567–76. doi: 10.1084/jem.190.4.567 10449527 PMC2195601

[28] Zucchi R Ronca-Testoni S . The sarcoplasmic reticulum Ca2+ channel/Ryanodine receptor: modulation by endogenous effectors, drugs and disease states. Pharmacol Rev. (1997) 49:1–38. doi: 10.1016/s0031-6997(24)01312-7 9085308

[29] Ducreux S Zorzato F Ferreiro A Jungbluth H Muntoni F Müller CR . Functional properties of ryanodine receptors carrying three amino acid substitutions identified in patients affected by multi-minicore disease and central core disease, expressed in immortalized lymphocytes. Biochem J. (2006) 395:259–66. doi: 10.1042/bj20051282 16372898 PMC1422771

[30] Gafni J Munsch JA Lam TH Catlin MC Costa LG Molinski TF . Xestospongins: potent membrane permeable blockers of the inositol 1,4,5-trisphosphate receptor. Neuron. (1997) 19:723–33. doi: 10.1016/s0896-6273(00)80384-0 9331361

[31] Ciepielewski ZM Stojek W Glac W Wrona D . Restraint effects on stress-related hormones and blood natural killer cell cytotoxicity in pigs with mutated ryanodine receptor. Domest Anim Endocrinol. (2013) 44:195–203. doi: 10.1016/j.domaniend.2013.02.003 23571007

[32] Sei Y Gallagher KL Basile A . Skeletal muscle type ryanodine receptor is involved in calcium signaling in human B lymphocytes. J Biol Chem. (1999) 274:5995–6002. doi: 10.1074/jbc.274.9.5995 10026226

[33] Diercks BP Werner R Weidemüller P Czarniak F Hernandez L Lehmann C . ORAI1, STIM1/2 and RYR1 shape subsecond Ca2+ microdomains upon T cell activation. Sci Signal. (2018) 11:eaat0358. doi: 10.1126/scisignal.aat0358 30563862 PMC6728084

[34] Nawata T Sakai H Honda T Otsuka M Fujita H Uchinoumi H . Dantrolene a stabilizer of the ryanodine receptor, prevents collagen-induced arthritis. Biochem Biophys Res Commun. (2022) 624:141–5. doi: 10.1016/j.bbrc.2022.07.111 35940127

[35] Yang L Dedkova EN Allen PD Saleet J Fomina AF . T lymphocytes from Malignant hyperthermia-susceptible mice display aberrations in intracellular calcium signaling and mitochondrial function. Cell calcium. (2021) 93:102325. doi: 10.1016/j.ceca.2020.102325 33310301 PMC7840221

[36] Klein SL Flanagan KL . Sex differences in immune responses. Nat Rev Immunol. (2016) 16:626–38. doi: 10.1038/nri.2016.90 27546235

[37] Lawrence MG Woodfolk JA Schuyler AJ Stillman LC Chapman MD Platts-Mills TAE . Half-life of IgE in serum and skin: consequences for anti-IgE therapy in patients with allergic diseases. J Allergy Clin Immunol. (2018) 139:422–8. doi: 10.1016/j.jaci.2016.04.056 27496596 PMC5405770

[38] Kushner I . Regulation of the acute phase response by cytokines. Perspect Biol Med. (1993) 36:611–22. doi: 10.1353/pbm.1993.0004 8361844

[39] Ogulur I Mitamura Y Yazici D Pat V Ardicli S Li M . Type 2 immunity in allergic diseases. Cell Mol Immunol. (2025) 22:211–42. doi: 10.1038/s41423-025-01261-2 39962262 PMC11868591

[40] Chung F . Anti-inflammatory cytokines in asthma and allergy: interleukin-10, interleukin-12, interferon-g. Mediators Inflammation. (2001) 10:51–9. doi: 10.1080/09629350120054518 11405550 PMC1781697

[41] Kanegane H Wakiguchi H Kanegane C Kurashige T Tosato G . Viral interleukin-10 in chronic active Epstein-Barr infection. J Infect Dis. (1997) 176:254–7. doi: 10.1086/517260 9207376

[42] Boonpiyathad T Satitsuksanoa P Akdis M Akdis CA . IL-10 producing T and B cells in allergy. Semin Immunol. (2019) 44:101326. doi: 10.1016/j.smim.2019.101326 31711770

[43] Moore KW de Waal Malefyt R Coffman RL O'Garra A . Interluein-10 and the interleukin-10 receptor. Annu Rev Immunol. (2001) 19:683–765. doi: 10.1146/annurev.immunol.19.1.683 11244051

[44] Maynard CL Weaver CT . Diversity in the contribution of interleukin-10 to T-cell-mediated immune regulation. Immunol Rev. (2008) 226:219–33. doi: 10.1111/j.1600-065x.2008.00711.x 19161427 PMC2630587

[45] Treves S Jungbluth H Voermans N Muntoni F Zorzato F . Ca2+ handling abnormalities in early-onset muscle diseases: novel concepts and perspectives. Sem Cell Dev Biol. (2017) 64:201. doi: 10.1016/j.semcdb.2016.07.017 27427513

[46] Hunter CA Jones SA . IL-6 as a keystone cytokine in health and disease. Nat Immunol. (2015) 16:448–57. doi: 10.1038/ni.3153 25898198

[47] Luan D Dadpey B Zaid J Bridge-Comer PE DeLuca JH Xia W . Adipocyte-secreted IL-6 sensitizes macrophages to IL-4 signaling. Diabetes. (2022) 72:367–74. doi: 10.2337/figshare.21637169.v1 PMC9935493 36449000

[48] Pederson BK Fischer CP . Beneficial health effects of exercise- the role of IL-6 as a myokine. Trends Pharmacol Sci. (2007) 28:152–6. doi: 10.1016/j.tips.2007.02.002 17331593

[49] Ducreux S Zorzato F Müller C Sewry C Muntroni F Quinlivan R . Effect of ryanodine receptor mutations on interleukin-6 release and intracellular calcium homeostasis in human myotubes from Malignant hyperthermia-susceptible individuals and patients affected by central core disease. J Biol Chem. (2004) 279:43838–64. doi: 10.1074/jbc.m403612200 15299003

[50] Riazi S van den Bersselaar LR Islander G Heytens L Snoeck MM Bjorksten A . Pre-operative exercise and pyrexia as modifying factors in Malignant hyperthermia (MH). Neuromuscul Disord. (2022) 32:628–34. doi: 10.1016/j.nmd.2022.06.003 35738978

[51] Natarajan K Sahoo NC Rao KVS . Signal thresholds and modular synergy during expression of costimulatory molecules in B lymphocytes. J Immunol. (2001) 167:114–22. doi: 10.4049/jimmunol.167.1.114 11418639

[52] Good KL Avery DT Tangye SG . Resting human memory B cells are intrinsically programmed for enhanced survival and responsiveness to diverse stimuli compared to naïve B cells. J Immunol. (2009) 182:890–901. doi: 10.4049/jimmunol.182.2.890 19124732

[53] Halliday N Williams C Kennedy A Waters E Pesenacker AM Soskic B . CD86 is a selective CD28 ligand supporting FoxP3+ regulatory T cell homeostasis in the presence of high levels of CTLA-4. Front Immunol. (2020) 11:600000. doi: 10.3389/fimmu.2020.600000 33363541 PMC7753196

[54] Caux C Vanbervliet B Massacrier C Azuma M Okumura K Lanier LL . B70/B7-2 is identical to CD86 and is the major functional ligand for CD28 expressed on human dendritic cells. J Exp Med. (1994) 180:1841–7. doi: 10.1084/jem.180.5.1841 7525840 PMC2191743

[55] Blair PJ Riley JL Harlan DM Abe R Tadaki DK Hoffmann SC . CD40 ligand (CD154) triggers a short-term CD4(+) T cell activation response that results in secretion of immunomodulatory cytokines and apoptosis. J Exp Med. (2000) 191:651–60. doi: 10.1084/jem.191.4.651 10684857 PMC2195831

[56] Suzuki M Yokota M Matsumoto Ozaki S . Synergistic effects of CD40 and CD86 silencing in dendritic cells on the control of allergic diseases. Int J Arch Allergy Immunol. (2018) 177:87–96. doi: 10.1159/000489862 30001545

[57] Silva LA Silva AF Ribeiro AC Silva AO Vieira FA Segundo GRS . Adult food allergy prevalence: reducing questionnaire bias. Int Arch Allergy Immunol. (2016) 171:261–4. doi: 10.1159/000453036 28049191

[58] Harris A Taylor R Thielke R Payne J Gonzalez N Conde JG . Research electronic data capture (REDCap) – A metadata-driven methodology and workflow process for providing translational research informatics support. J BioMed Inform. (2009) 42:377–81. doi: 10.1016/j.jbi.2008.08.010 18929686 PMC2700030

[59] Harris PA Taylor R Minor BL Elliott V Fernandez M O'Neal L . The REDCap consortium: Building an international community of software partners. J BioMed Inform. (2019) 95:103208. doi: 10.1016/j.jbi.2019.103208 31078660 PMC7254481

