# Supplementary material for: RYR1 mutations linked to malignant hyperthermia susceptibility are associated with phenotypic changes in human B-lymphocytes
Source: Front Immunol. 2026 Jul 27;17:1876709. doi: 10.3389/fimmu.2026.1876709 (PMC13453734; doi:10.3389/fimmu.2026.1876709)
Supplement: Supplementary file 1 [file DataSheet1.docx]

**Figure 1A / Figure 1B:**

| Allergy Comparison: Family MHN vs MHS |  |  |  |  |  |  |  |
| --- | --- | --- | --- | --- | --- | --- | --- |
| Allergy Type | HC (n=31) | MHS (n=70) | Brazilian  Avg (n=2003) | P-value (HC vs MHS) | FDR q-value  (HC vs MHS) | P-value  (MHS vs Brazilian Avg) | FDR q-value  (MHS vs Brazilian Avg) |
| Overall | 16 (51.6%) | 33 (47.1%) | 748 (37.3%) | 0.829 | 1 | 0.103 | 0.129 |
| Rhinitis | 8 (25.8%) | 27 (38.6%) | 518 (25.9%) | 0.261 | 0.653 | 0.026* | 0.129 |
| Asthma | 2 (6.5%) | 7 (10.0%) | 99 (4.9%) | 0.718 | 1 | 0.087 | 0.129 |
| Eczema | 4 (12.9%) | 2 (2.9%) | 185 (9.2%) | 0.07 | 0.35 | 0.085 | 0.129 |
| Food Allergy | 6 (19.7%) | 9 (12.9%) | 208 (10.4%) | 1 | 1 | 0.549 | 0.549 |

**Figure 1C:**

| Infection Type | HC (n=31) | MHS (n=70) | P-value (HC vs MHS) |
| --- | --- | --- | --- |
| Influenza Virus Infection | 89.5 | 77.8 |  |
| COVID-19 Infection | 77.4 | 57.1 |  |

**Figure 3A:**

| Cell Type | HC (n) | HC Mean ± SD | MHS (n) | MHS Mean ± SD | P-value |
| --- | --- | --- | --- | --- | --- |
| T CELLS | 14 | 18.32 ± 10.51 | 10 | 22.84 ± 8.21 | 0.2693 |
| B CELLS | 14 | 5.65 ± 3.73 | 10 | 3.86 ± 2.01 | 0.183 |
| NK CELLS | 14 | 37.01 ± 10.29 | 10 | 35.98 ± 17.54 | 0.8582 |
| MYELOID CELLS | 14 | 17.17 ± 11.93 | 10 | 12.10 ± 12.55 | 0.3256 |

**Figure 3B:**

| B Cell Subtype | HC (Mean ± SD) | MHS (Mean ± SD) | Raw p-value | BH FDR q-value (m=6) |
| --- | --- | --- | --- | --- |
| Naive B Cells | 43.35 ± 6.80 | 37.96 ± 9.08 | 0.1101 | 0.3559 |
| Transitional B Cells | 1.54 ± 1.30 | 1.61 ± 1.86 | 0.9094 | 0.9094 |
| Non-switched Memory | 15.12 ± 6.50 | 19.66 ± 9.23 | 0.1697 | 0.3559 |
| Switched Memory | 12.33 ± 3.48 | 14.22 ± 4.89 | 0.2795 | 0.4192 |
| Effector B cells | 4.12 ± 1.79 | 5.67 ± 3.63 | 0.178 | 0.3559 |
| Breg Cells | 2.72 ± 2.41 | 3.90 ± 3.92 | 0.3734 | 0.4481 |

**Figure 3C:**

| B Cell Subtype | HC (Mean ± SD) | MHS (Mean ± SD) | Raw p-value | BH FDR q-value (m=7) |
| --- | --- | --- | --- | --- |
| Whole B Cells | 18.40 ± 5.15 | 29.15 ± 9.52 | 0.0017 | 0.006 |
| Transitional B Cells | 22.11 ± 5.45 | 31.77 ± 8.74 | 0.003 | 0.0069 |
| Naive B Cells | 16.73 ± 5.44 | 23.83 ± 8.53 | 0.0207 | 0.029 |
| Non-switched Memory | 23.74 ± 6.68 | 33.46 ± 11.83 | 0.0176 | 0.029 |
| Switched Memory | 19.05 ± 5.08 | 30.02 ± 9.14 | 0.0011 | 0.006 |
| Effector B cells | 25.11 ± 10.64 | 37.14 ± 16.12 | 0.0379 | 0.0443 |
| Breg Cells | 32.04 ± 15.51 | 40.47 ± 13.43 | 0.1796 | 0.1796 |

**Figure 3D:**

| B Cell Subtype | HC (Mean ± SD) | MHS (Mean ± SD) | Raw p-value | BH FDR q-value (m=7) |
| --- | --- | --- | --- | --- |
| Whole B Cells | 3976.74 ± 362.37 | 4758.99 ± 521.53 | 0.0003 | 0.0018 |
| Transitional B Cells | 3337.96 ± 687.00 | 4012.64 ± 868.31 | 0.0449 | 0.0524 |
| Naive B Cells | 3775.30 ± 433.19 | 4580.14 ± 556.70 | 0.0006 | 0.0022 |
| Non-switched Memory | 3831.47 ± 525.30 | 4754.49 ± 733.27 | 0.0016 | 0.0024 |
| Switched Memory | 4309.48 ± 329.38 | 4945.82 ± 543.54 | 0.0017 | 0.0024 |
| Effector B cells | 2767.25 ± 360.58 | 3251.16 ± 273.74 | 0.0017 | 0.0024 |
| Breg Cells | 3820.23 ± 596.21 | 4068.18 ± 932.89 | 0.4346 | 0.4346 |

**Figure 4A:**

| Ig Type | n (HC) | Mean ± SD (HC) | n (MHS) | Mean ± SD (MHS) | Test | p-value |
| --- | --- | --- | --- | --- | --- | --- |
| IgM | 26 | 1.18 ± 0.47 | 11 | 0.84 ± 0.34 | Welch's t-test | 0.022 |
| IgA | 26 | 2.18 ± 1.01 | 11 | 1.78 ± 1.18 | Welch's t-test | 0.344 |
| IgG | 26 | 10.84 ± 2.19 | 11 | 9.98 ± 2.67 | Welch's t-test | 0.361 |
| IgG1 | 26 | 7.20 ± 1.56 | 11 | 6.47 ± 2.07 | Welch's t-test | 0.304 |
| IgG2 | 26 | 3.07 ± 1.31 | 11 | 3.11 ± 1.04 | Welch's t-test | 0.927 |
| IgG3 | 26 | 0.32 ± 0.12 | 11 | 0.39 ± 0.17 | Welch's t-test | 0.231 |
| IgG4 | 26 | 0.72 ± 0.64 | 11 | 0.52 ± 0.22 | Welch's t-test | 0.19 |

**Figure 4A (IgA2 panel):**

| Group | n | Mean ± SD | Median (IQR) | p-value | Statistical Test |
| --- | --- | --- | --- | --- | --- |
| HC (Healthy Control) | 24 | 14016.45 ± 168.76.06 | 9561.46 (384.38–18337.89) | 0.921 | Mann-Whitney U Test |
| MHS (MHS Patients) | 9 | 14930.13 ± 15972.21 | 8723.08 (62.82–22158.97) |  |  |

**Figure 4B:**

| Group | Valid Samples (n) | Excluded Samples  (no CMV IgG) | Mean ± SD | Statistical Test | P-value (HC vs MHS) |
| --- | --- | --- | --- | --- | --- |
| HC (Healthy Control) | 13 | 4 | 0.67 ± 0.20 | Welch's t-test | 0.8983 |
| MHS (MHS Patients) | 6 | 2 | 0.67 ± 0.07 |  |  |

**Figure 4C:**

| Cytokine | Group | n | Mean ± SD | Median (IQR) | p-value | Statistical Test |
| --- | --- | --- | --- | --- | --- | --- |
| BAFF | MHN | 21 | 406.99 ± 562.23 | 241.50 (57.50–378.38) | 0.1414 | Mann-Whitney U Test |
|  | MHS | 6 | 803.85 ± 538.40 | 987.13 (476.19–1057.91) |  |  |
| IL-6 | MHN | 21 | 0.42 ± 0.92 | 0.00 (0.00–0.27) | 0.005 |  |
|  | MHS | 6 | 2.50 ± 2.29 | 2.19 (1.01–3.24) |  |  |
| IL-4 | MHN | 21 | 1.61 ± 3.19 | 0.71 (0.62–1.09) | 0.0063 |  |
|  | MHS | 6 | 2.20 ± 1.31 | 1.72 (1.61–2.64) |  |  |
| IL-10 | MHN | 21 | 0.53 ± 1.48 | 0.00 (0.00–0.00) | 0.0004 |  |
|  | MHS | 6 | 8.89 ± 17.20 | 1.45 (0.73–4.64) |  |  |

**Figure 5B:**

| Comparison | N (HC) | N (MHS) | Mean ± SD (HC) | Mean ± SD (MHS) | P-value | Significance | Statistic Test |
| --- | --- | --- | --- | --- | --- | --- | --- |
| HC vs MHS | 9 | 8 | 632.60 ± 41.46 | 572.80 ± 19.61 | 0.001 | *** | Mann-Whitney U |

**Figure 5C:**

| Figure 5C | | | | | |
| --- | --- | --- | --- | --- | --- |
| Group | n | Mean ± SD | Median (IQR) | p-value | Statistical Test |
| HC (Healthy Control) | 9 | 1.00 ± 0.33 | 0.96 (0.82–1.08) | 0.246 | Mann-Whitney U Test |
| MHS (MHS Patients) | 8 | 0.82 ± 0.59 | 0.76 (0.52–0.90) |  |  |

**Figure 5D:**

| Group | n | Mean ± SD | Median (IQR) | p value | Statistical Test |
| --- | --- | --- | --- | --- | --- |
| HC (Healthy Control) | 9 | 1.38 ± 0.22 | 1.31 (1.29–1.56) | 0.7975 | Mann-Whitney U Test |
| MHS (MHS Patients) | 8 | 1.39 ± 0.40 | 1.26 (1.16–1.61) |  |  |

**Figure 5E:**

| Group | n | Mean ± SD | Median (IQR) | p-value | Statistical Test |
| --- | --- | --- | --- | --- | --- |
| HC (Healthy Control) | 9 | 6362.75 ± 2898.43 | 6062.33 (4539.66–7985.87) | 0.0016 | Mann-Whitney U Test |
| MHS (MHS Patients) | 8 | 1914.11 ± 1242.27 | 1726.10 (1091.39–2895.98) |  |  |

**Figure 6A:**

| Group | n | Mean ± SD | p-value | Statistical Test |
| --- | --- | --- | --- | --- |
| HC (Healthy Control) | 9 | 1.00 ± 0.33 | 0.246 | Mann-Whitney U Test |
| MHS (MHS Patients) | 8 | 0.82 ± 0.59 |  |  |

**Figure 6B:**

| Time | Group | n | Mean ± SD | Comparison | Raw p-value | Bonferroni p-value | Significance |
| --- | --- | --- | --- | --- | --- | --- | --- |
| 0 h | Medium only | 9 | 1.00 ± 0.00 | – | – | – | – |
| 0 h | 10 mM Caffeine | 9 | 1.00 ± 0.00 | – | – | – | – |
| 0 h | Caff + Tetra | 9 | 1.00 ± 0.00 | – | – | – | – |
| 24 h | Medium only | 9 | 0.66 ± 0.37 | – | – | – | – |
| 24 h | 10 mM Caffeine | 9 | 1.51 ± 0.75 | vs Medium | 0.0134 | 0.1206 | ns |
| 24 h | Caff + Tetra | 9 | 1.79 ± 1.23 | vs Caffeine | 0.9296 | 1 | ns |
| 48 h | Medium only | 9 | 1.07 ± 0.29 | – | – | – | – |
| 48 h | 10 mM Caffeine | 9 | 1.93 ± 1.08 | vs Medium | 0.0774 | 0.6966 | ns |
| 48 h | Caff + Tetra | 9 | 1.92 ± 1.58 | vs Caffeine | 0.5365 | 1 | ns |
| 72 h | Medium only | 9 | 1.46 ± 0.50 | – | – | – | – |
| 72 h | 10 mM Caffeine | 9 | 1.48 ± 0.76 | vs Medium | 1 | 1 | ns |
| 72 h | Caff + Tetra | 9 | 1.53 ± 1.60 | vs Caffeine | 0.3314 | 1 | ns |

**Figure 6C:**

| Time | Group | n | Mean ± SD | Comparison | Raw p-value | Bonferroni p-value | Significance |
| --- | --- | --- | --- | --- | --- | --- | --- |
| 0 h | Medium only | 8 | 1.00 ± 0.00 | – | – | – | – |
| 0 h | 10 mM Caffeine | 8 | 1.00 ± 0.00 | – | – | – | – |
| 0 h | Caff + Tetra | 8 | 1.00 ± 0.00 | – | – | – | – |
| 24 h | Medium only | 8 | 1.00 ± 0.56 | – | – | – | – |
| 24 h | 10 mM Caffeine | 8 | 3.47 ± 2.16 | vs Medium | 0.0019 | 0.0171 | * |
| 24 h | Caff + Tetra | 8 | 1.77 ± 1.43 | vs Caffeine | 0.0379 | 0.3411 | ns |
| 48 h | Medium only | 8 | 1.38 ± 1.06 | – | – | – | – |
| 48 h | 10 mM Caffeine | 8 | 5.69 ± 4.03 | vs Medium | 0.0019 | 0.0171 | * |
| 48 h | Caff + Tetra | 8 | 2.36 ± 1.81 | vs Caffeine | 0.0499 | 0.4491 | ns |
| 72 h | Medium only | 8 | 1.66 ± 1.31 | – | – | – | – |
| 72 h | 10 mM Caffeine | 8 | 4.32 ± 2.46 | vs Medium | 0.0047 | 0.0423 | * |
| 72 h | Caff + Tetra | 8 | 2.50 ± 1.76 | vs Caffeine | 0.0499 | 0.4491 | ns |

**Supplementary Figure 2:**

| Surface  Marker | p_value | Bonferroni (m=13) | Holm (m=13) | BH FDR q-value (m=13) |
| --- | --- | --- | --- | --- |
| IgA | 0.05319108 | 0.69148398 | 0.58510183 | 0.23049466 |
| IgG | 0.20387891 | 1 | 1 | 0.52026158 |
| CD83 | 0.23802939 | 1 | 1 | 0.52026158 |
| IgM | 0.24012073 | 1 | 1 | 0.52026158 |
| PD-L1 | 0.302613 | 1 | 1 | 0.52919225 |
| MHC-II | 0.32565677 | 1 | 1 | 0.52919225 |
| CD39 | 0.41495831 | 1 | 1 | 0.57530562 |
| CD85j | 0.45731833 | 1 | 1 | 0.57530562 |
| IgD | 0.48679706 | 1 | 1 | 0.57530562 |
| BAFFR | 0.64871182 | 1 | 1 | 0.70277114 |
| CD71 | 0.94263462 | 1 | 1 | 0.94263462 |

**Supplementary Figure 3:**

| Marker | Group | n | Mean ± SD | p-value |
| --- | --- | --- | --- | --- |
| CD86 | HC | 14 | 1.000 ± 0.179 | 0.2904 |
|  | MHS | 10 | 1.236 ± 0.224 | - |
| CD40 | HC | 14 | 1.000 ± 0.328 | 0.7678 |
|  | MHS | 10 | 0.968 ± 0.187 | - |
| CD83 | HC | 14 | 1.0000 ± 0.6559 | 0.4431 |
|  | MHS | 10 | 1.5479 ± 1.4880 | - |
| MHCII | HC | 14 | 1.0000 ± 0.5100 | 0.5843 |
|  | MHS | 10 | 1.1359 ± 0.7719 | - |

**Supplementary Figure 4:**

| Cytokine | Group | n | Mean ± SD | Median (IQR) | p-value | Statistical Test |
| --- | --- | --- | --- | --- | --- | --- |
| IL-6 | MHN | 21 | 0.42 ± 0.92 | 0.00 (0.00–0.27) | 0.005 | Mann-Whitney U Test |
|  | MHS | 6 | 2.50 ± 2.29 | 2.19 (1.01–3.24) |  |  |
| IL-4 | MHN | 21 | 1.61 ± 3.19 | 0.71 (0.62–1.09) | 0.0063 |  |
|  | MHS | 6 | 2.20 ± 1.31 | 1.72 (1.61–2.64) |  |  |
| IL-10 | MHN | 21 | 0.53 ± 1.48 | 0.00 (0.00–0.00) | 0.0004 |  |
|  | MHS | 6 | 8.89 ± 17.20 | 1.45 (0.73–4.64) |  |  |

**Supplementary Figure 5:**

|  | Group | n | Mean ± SD | Median (IQR) | p value | Statistical Test |
| --- | --- | --- | --- | --- | --- | --- |
| IgA2 | Brasilian Control Cohort | 10 | 20,746.85 ± 18,768.48 | 15,592.01 (3,433.01–38,264.74) | 0.2653 | Mann-Whitney U Test |
|  | Basel Control Cohort | 14 | 6,658.24 ± 7,798.94 | 1,725.63 (430.00–13,501.95) |  |  |
| IL-10 | Brasilian Control Cohort | 7 | 0.18 ± 0.47 | 0.00 (0.00–0.00) | 0.8404 | Mann-Whitney U Test |
|  | Basel Control Cohort | 14 | 0.70 ± 1.78 | 0.00 (0.00–0.00) |  |  |
| IL-6 | Brasilian Control Cohort | 7 | 0.48 ± 0.85 | 0.18 (0.00–0.41) | 0.3667 | Mann-Whitney U Test |
|  | Basel Control Cohort | 14 | 0.39 ± 0.98 | 0.00 (0.00–0.20) |  |  |
| IL-4 | Brasilian Control Cohort | 7 | 0.92 ± 0.55 | 0.71 (0.65–0.82) | >0.9999 | Mann-Whitney U Test |
|  | Basel Control Cohort | 14 | 1.96 ± 3.89 | 0.72 (0.62–1.19) |  |  |
| BAFF | Brasilian Control Cohort | 7 | 493.02 ± 578.73 | 269.63 (239.94–394.31) | 0.2211 | Mann-Whitney U Test |
|  | Basel Control Cohort | 14 | 363.97 ± 570.75 | 136.88 (0.00–352.50) |  |  |

**Supplementary Figure 6:**

|  | Group | n | Mean ± SD | Median (IQR) | p value | Statistical Test |
| --- | --- | --- | --- | --- | --- | --- |
| Intracellular IL-10 | HC | 8 | 120.19 ± 56.87 | 108.46 (91.97–122.11) | 0.1709 | Mann-Whitney U test |
|  | MHS | 5 | 80.11 ± 33.93 | 95.28 (78.51–95.81) |  |  |
| Intracellular IL-12 | HC | 8 | 24.42 ± 5.97 | 23.68 (21.66–29.21) | 0.0617 | Mann-Whitney U test |
|  | MHS | 5 | 14.64 ± 8.47 | 17.52 (14.39–19.72) |  |  |

**Supplementary Figure 7A:**

|  | HC (+10mM Caffeine) Mean ± SD (n=9) | MHS (+10mM Caffeine) Mean ± SD (n=8) | Mann-Whitney U p-value | Significance |
| --- | --- | --- | --- | --- |
| CD83 |  |  |  |  |
| 24h | 0.8200 ± 1.7272 | 0.7581 ± 0.4009 | 0.4234 | ns |
| 48h | 1.0423 ± 0.3416 | 0.8539 ± 0.5729 | 0.4234 | ns |
| 72h | 0.9988 ± 0.3762 | 0.6809 ± 0.2740 | 0.1388 | ns |

**Supplementary Figure 7B:**

|  | HC (+10mM Caffeine) Mean ± SD (n=9) | MHS (+10mM Caffeine) Mean ± SD (n=8) | Mann-Whitney U p-value | Significance |
| --- | --- | --- | --- | --- |
| CD40 |  |  |  |  |
| 24h | 0.7632 ± 0.2568 | 1.4386 ± 1.3177 | 0.2359 | ns |
| 48h | 0.9669 ± 0.3898 | 2.3172 ± 2.9704 | 0.3704 | ns |
| 72h | 0.7589 ± 0.4575 | 1.5048 ± 1.8660 | 0.9626 | ns |

**Supplementary Figure 7C:**

| Cell Line | Time | Comparison | Experimental Replicates (n) | P-value | Bonferroni p-value | Significance |
| --- | --- | --- | --- | --- | --- | --- |
| HC3 | 0h | All Comparisons | 3 | N/A | - | ns |
| HC3 | 24h | Caffeine vs Caff+Xesto | 3 | 0.0593 | 0.5337 | ns |
| HC3 | 24h | Caffeine vs Xesto | 3 | 0.0014 | 0.0126 | * |
| HC3 | 24h | Caff+Xesto vs Xesto | 3 | 0.0244 | 0.2196 | ns |
| HC3 | 48h | Caffeine vs Caff+ Xesto | 3 | 1 | 1 | ns |
| HC3 | 48h | Caffeine vs Xesto | 3 | 0.0044 | 0.0396 | * |
| HC3 | 48h | Caff+Xesto vs Xesto | 3 | 0.0044 | 0.0396 | * |
| HC3 | 72h | Caffeine vs Caff+Xesto | 3 | 0.9518 | 1 | ns |
| HC3 | 72h | Caffeine vs Xesto | 3 | 0.0021 | 0.0189 | * |
| HC3 | 72h | Caff+Xesto vs Xesto | 3 | 0.0028 | 0.0252 | * |

**Supplementary Figure 7D:**

| Cell Line | Time | Comparison | Experimental Replicates (n) | P-value | Bonferroni p-value | Significance |
| --- | --- | --- | --- | --- | --- | --- |
| MHS3 | 0h | All Comparisons | 3 | N/A | - | ns |
| MHS3 | 24h | Caffeine vs Caff+Xesto | 3 | 0.0099 | 0.0891 | ns |
| MHS3 | 24h | Caffeine vs Xesto | 3 | 0 | <0.0001 | *** |
| MHS3 | 24h | Caff+Xesto vs Xesto | 3 | 0 | <0.0001 | *** |
| MHS3 | 48h | Caffeine vs Caff+Xesto | 3 | 0.4755 | 1 | ns |
| MHS3 | 48h | Caffeine vs Xesto | 3 | 0 | <0.0001 | *** |
| MHS3 | 48h | Caff+Xesto vs Xesto | 3 | 0 | <0.0001 | *** |
| MHS3 | 72h | Caffeine vs Caff+Xesto | 3 | 0.167 | 1 | ns |
| MHS3 | 72h | Caffeine vs Xesto | 3 | 0 | <0.0001 | *** |
| MHS3 | 72h | Caff+Xesto vs Xesto | 3 | 0 | <0.0001 | *** |
