# Supplementary material for: RYR1 mutations linked to malignant hyperthermia susceptibility are associated with phenotypic changes in human B-lymphocytes
Source: Front Immunol. 2026 Jul 27;17:1876709. doi: 10.3389/fimmu.2026.1876709 (PMC13453734; doi:10.3389/fimmu.2026.1876709)
Supplement: Supplementary file 2 [file SupplementaryFile1.docx]

Supplementary Material

# 1 Supplementary Figures

**Supplementary Figure 1.** **Flow cytometry gating strategy and surface expression of CD86 and CD40 on B-cell subsets. A.** Gating strategy for major peripheral blood immune cell populations. Doublets were excluded based on FSC-A versus FSC-H, followed by gating on live cells using viability dye exclusion. Total cells were identified and CD45⁺ leukocytes selected. Major immune populations were defined as CD3⁺ T cells, CD19⁺ B cells, CD3⁻CD56⁺ NK cells, and CD14⁺ myeloid cells. **B.** Detailed gating strategy used to define B-cell subsets within the CD19⁺ B-cell compartment. B cells were first identified as CD19⁺CD20⁺ cells. Within this population, cells were separated according to CD21 and CD38 expression to distinguish CD38⁺CD21⁻ effector B cells from the remaining common B-cell population. The common B-cell population was then further subdivided based on IgD and CD27 expression into naïve/transitional B cells, non-switched memory B cells, switched memory B cells, and double-negative B cells. Naïve and transitional B cells were subsequently resolved from the IgD⁺CD27⁻ compartment using CD38 and CD10 expression, with transitional B cells defined as CD38^high^/CD10⁺ and naïve B cells defined as CD38^low/−^CD10⁻. In parallel, regulatory B cells were identified within the B-cell compartment based on high CD24 and CD38 expression, and further assessed for CD10 expression. **C. and D.** Representative overlay histograms showing surface expression of CD86 (C) and CD40 (D) on total B cells and the indicated subsets, including naïve, transitional, non-switched memory, switched memory, effector, and regulatory B cells. Data represent one healthy control (HC7, blue) and one MHS donor (MHS4, red). The x-axis indicates fluorescence intensity and the y-axis indicates relative cell frequency (FlowJo modal display setting).

**Supplementary Figure 2. Surface marker expression in B-cells from Healthy Controls (HC) and Malignant Hyperthermia Susceptible /MHS) individuals.** **Quantitative comparison of B-cell subset frequencies and surface marker expression.** Bar graphs indicate the relative Mean Fluorescence Intensity (MFI) of the indicated 11 surface markers in cells from 14 HC (white bars, n=14) and MHS (grey bars, n=10). Bars represent mean (±s.d.) and each symbol represents one individual. Statistical comparisons between HC and MHS were performed using Welch’s t test. No significant differences were detected between the groups.

**Supplementary Figure 3: Maturation marker surface expression on the myeloid cell compartment of Healthy Controls and Malignant Hyperthermia Susceptible individuals.** Quantitative comparison of CD86, CD40, CD83, and MHC class II (MHC-II) surface expression on myeloid cells (CD45+ CD56- MHC-II^high^ CD14+). Bar graphs indicate the normalized mean (± s.d.) fluorescence intensity (MFI) of the indicated surface markers in cells from HC (white bars, circles, n = 14) and MHS individuals (grey bars, squares, n = 10). Each symbol represents one individual. Statistical comparisons between HC and MHS were performed using Welch’s t-test. No significant differences were detected between the two groups.

**Supplementary Figure 4. Heat map of IL-4, IL-6 and IL-10 concentrations in the serum of HC and MHS individuals.** The concentration of each cytokine in the serum of MHS (n=6) and HC (n= 21 of which n=7 were HC from the Brazilian cohort and n=14 were HC from the Basel Blood donor clinic (C1-C15). The heatmap shows the cytokine concentration in the serum of each subject determined by ELISA experiments performed in technical duplicates. The concentration of each cytokine was determined using a standard curve generated using cytokine standards included in the kits. Color scale: range from 0 pg/ml (dark blue) to 6 pg/ml (yellow) and all values > 6 pg/ml are shown as orange.

**Supplementary Figure 5: Serum concentration of IgA2, BAFF, IL-4, IL-6 and IL-10 in the serum of the healthy control cohort from Brazil and Basel**. Bar graphs show the mean (±s.d.) [IgA2] and cytokine concentration in serum samples from the Brazilian HC cohort (empty white bars) and the Basel blood bank cohort (striped bars). Each symbol represents results from one individual. ELISA experiments were performed in technical duplicates. No significant differences were detected between the two groups.

**Supplementary Figure 6. The intracellular concentrations of IL-10 and IL-12 are similar in EBV-transformed B cells from MHS and HC.** Concentration of IL-10 and IL-12 were determined 1x10^6^ lysed EBV-immortalized cells from healthy control (HC white bar, each circle represents one individual n=8) and Malignant Hyperthermia Susceptible individuals (MHS grey bar, each square represents one individual n=5). Each data point is the mean of at least three independent ELISA experiments performed in technical duplicates. Data are presented as mean ± s.d

**Supplementary Figure 7. Caffeine does not stimulate CD83 and CD40 surface expression on EBV-immortalized B cells, and CD86 surface expression by caffeine is not affected by Xestospongin C. A** and **B.**  Bar graph showing the time-course of CD83 (panel A) and CD40 (panel B) surface expression in EBV-immortalized B cells from the HC cohort (white bars, n = 9) and MHS cohort (grey bars, n = 8) following stimulation with 10 mM caffeine, at t=0, t=24, t=48, and t= 72 hours post-stimulation. **C.** and **D.** Bar graphs showing the time-dependent fold change in CD86 surface expression (at t=0, t=24, t= 48, and t=72 hours) in cell line HC3 (panel C) and MHS3 (panel D). White bars, cells incubated with 10 mM caffeine; light grey bars, cells incubated in medium containing 10 mM caffeine plus 2 µM Xestospongin C; dark grey bars cells incubated in medium containing 2 µM Xestospongin C. The bars shown in panels C and D are the mean (±s.d.) of 3 independent experiments. The CD86 surface expression levels presented in panels C and D were normalized to the MFI observed at t=0. Statistical comparisons between HC and MHS groups for CD83 and CD40 (panels A and B, respectively) were performed using the Mann-Whitney U test. Statistical comparisons evaluating the effects of treatment conditions over time in individual cell lines (panels C and D) were performed using a two-way ANOVA followed by the Bonferroni Post hoc test. *p<0.05, ***p < 0.001.
