# Supplementary material for: RYR1 mutations linked to malignant hyperthermia susceptibility are associated with phenotypic changes in human B-lymphocytes
Source: Front Immunol. 2026 Jul 27;17:1876709. doi: 10.3389/fimmu.2026.1876709 (PMC13453734; doi:10.3389/fimmu.2026.1876709)
Supplement: Supplementary file 3 [file Table1.docx]

Supplementary Material

**Supplementary Table 1**: General characteristics of Brazilian cohorts enrolled in the questionnaire study.

| **Family N°** | **Patient Identification N°** | **Age/sex** | **IVCT^§^** | ***RYR1* mutation** | **Allergies** | **Influenza infection and symptoms^¶^** | **Covid-19 symptoms^¶^** |  |
| --- | --- | --- | --- | --- | --- | --- | --- | --- |
| 1 | 1.1 | 39/ F | MHS^ﬁ^ | unknown | None | no | no |  |
|  | 1.2 | 29/ F | MHS | unknown | None | yes (similar) | no |  |
| 2 | 2.1 | 66/ M | MHS | unknown | Rhin^†^ | yes (similar) | yes (severe) |  |
|  | 2.2 | 32/ F | MHN^#^ | **-** | None | yes  (mild) | yes  (mild) |  |
| 3 | 3.1 | 54/ F | MHS | c.1840 C>T  (p.R614C) | None | yes  (mild) | yes  (mild) |  |
|  | 3.2 | 38/ F | MHN | **-** | None | yes (similar) | yes (similar) |  |
| 4 | 4.1 | 25/ M | MHS | c.2366G>A  (p.R789Q) | None | yes (similar) | yes  (mild) |  |
| 5 | 5.1 | 42/ M | MHS | unknown | Rhin | no | yes (severe) |  |
| 6 | 6.1 | 53/ M | MHS | c.8197G>T  (p. G2733C) | None | no | no |  |
| 7 | 7.1 | 53/ F | MHN | **-** | Food, Eczema | yes  (mild) | yes  (mild) |  |
|  | 7.2 | 43/ F | MHS | unknown | None | yes  (mild) | yes (similar) |  |
|  | 7.3 | 27/ M | MHS | c.C7354G  (p.R2452G) | Rhin, Food, Eczema | yes (similar) | yes  (mild) |  |
|  | 7.4 | 39/ F | MHS | Unknown | Rhin, Asthma | no | yes (similar) |  |
| 8 | 8.1 | 48/ F | MHS | c. 1314G>A  (p.R3772W) | None | no | no |  |
| 9 | 9.1 | 34/ F | MHS | c.7300G>A  (p.G2434R) | None | yes  (mild) | no |  |
|  | 9.2 | 27/ F | MHS | c.7300G>A  (p.G2434R) | Asthma | yes (severe) | no |  |
| 10 | 10.1 | 38/ F | MHS | c.G10747C  (p.E3583Q) | None | yes (similar) | no |  |
| 11 | 11.1 | 61/ F | MHS | c.14918C>T  (p.P4973L) | Rhin | no | yes  (very mild) |  |
|  | 11.2 | 60/ M | MHS | unknown | None | yes (similar) | yes  (mild) |  |
| 12 | 12.1 | 34/ F | MHS | unknown | None | yes (similar) | no |  |
|  | 12.2 | 25/ F | MHN | **-** | Asthma | yes (similar) | yes  (mild) |  |
| 13 | 13.1 | 42/ M | MHS | unknown | None | no | no |  |
|  | 13.2 | 10/ F | MHS | unknown | None | no | no |  |
| 14 | 14.1 | 45/ F | MHS | c.C1840T  (p.R614C) | None | yes (similar) | yes  (very mild) |  |
| 15 | 15.1 | 36/ F | MHS | c.6544A>T  (p.I2182P) | Rhin, Eczema | yes (severe) | yes  (very severe) |  |
| 16 | 16.1 | 49/ F | MHS | unknown | Rhin, Food, Asthma | yes (similar) | yes (severe) |  |
| 17 | 17.1 | 39/ F | MHS | c.7300G>A  (p.G2434R) | None | yes (severe) | yes (similar) |  |
| 18 | 18.1 | 28/ M | MHS | unknown | Rhin | yes (similar) | yes (similar) |  |
|  | 18.2 | 24/ M | MHS | unknown | None | yes  (mild) | yes  (mild) |  |
| 19 | 19.1 | 51/ F | MHN | **-** | Eczema | yes  (mild) | no |  |
| 20 | 20.1 | 20/ F | MHN | **-** | None | yes (similar) | yes (similar) |  |
|  | 20.2 | 49/ F | MHS | unknown | None | yes  (mild) | yes (similar) |  |
| 21 | 21.1 | 35/ M | MHS | c.122T>C  (p.F41S) | Rhin | yes (similar) | yes  (mild) |  |
|  | 21.2 | 62/ F | MHN | **-** | Rhin | yes (similar) | no |  |
| 22 | 22.1 | 23/ M | MHS | unknown | Rhin | Yes  (hospital) | yes  (very mild) |  |
| 23 | 23.1 | 60/ M | MHS | unknown | None | yes  (mild) | no |  |
|  | 23.2 | 64/ F | MHS | unknown | Rhin | no | no |  |
| 24 | 24.1 | 52/ M | MHS | unknown | None | no | no |  |
| 25 | 25.1 | 41/ M | MHS | unknown | None | yes (similar) | yes (similar) |  |
| 26 | 26.1 | 71/ M | MHS | c.5999C>T  (p.S2000F) | Rhin | yes  (mild) | yes  (mild) |  |
| 27 | 27.1 | 56/ M | MHS | unknown | Asthma | no | no |  |
| 28 | 28.1 | 55/ F | MHN | **-** | Rhin | yes  (very mild) | yes (similar) |  |
| 29 | 29.1 | 30/ F | MHS | c.14545G>A  (p.V4849I) | Rhin, Food | yes  (very mild) | yes (similar) |  |
|  | 29.2 | 28/ M | MHS | unknown | Rhin, Food | yes  (mild) | yes (severe) |  |
|  | 29.3 | 55/ M | MHS | unknown | Rhin, Food | yes  (very mild) | yes  (very mild) |  |
|  | 29.4 | 54/ F | MHN | **-** | None | yes  (very mild) | yes (similar) |  |
| 30 | 30.1 | 55/ M | MHS | c.455C>A  (p.A152D) | None | no | yes (severe) |  |
| 31 | 31.1 | 43/ M | MHN | **-** | None | yes  (mild) | yes (similar) |  |
|  | 31.2 | 33/ F | MHN | **-** | None | yes (similar) | yes  (mild) |  |
|  | 31.3 | 13/ F | MHS | unknown | Food | yes  (mild) | no |  |
|  | 31.4 | 40/ M | MHS | unknown | None | yes (similar) | no |  |
| 32 | 32.1 | 46/ F | MHS | c.7358T>C  (p.I2453T) | Food | yes (severe) | no |  |
|  | 32.2 | 46/ M | MHN | **-** | None | yes  (very severe) | no |  |
|  | 32.3 | 24/ F | MHN | **-** | None | no | yes  (not specified) |  |
| 33 | 33.1 | 50/ M | MHS | unknown | Rhin | yes  (mild) | no |  |
| 34 | 34.1 | 34/ F | MHS | c.487C>T  (p.R163C) | None | yes (similar) | no |  |
|  | 34.2 | 38/ F | MHN | **-** | Food | yes  (very severe) | yes (severe) |  |
|  | 34.3 | 24/ F | MHN | **-** | None | yes (mild) | no |  |
| 35 | 35.1 | 28/ F | MHS | unknown | Rhin, Food | yes (mild) | yes (similar) |  |
| 36 | 36.1 | 58/ F | MHS | c.131G>A  (p.R44H) | None | yes (similar) | yes (severe) |  |
|  | 36.2 | 90/ M | MHS | unknown | None | yes (severe) | no |  |
|  | 36.3 | 39/ F | MHS | unknown | Rhin,  Asthma | yes (mild) | no |  |
|  | 36.4 | 66/ F | MHS | c.131G>A  (p.R44H) | None | yes  (very mild) | no |  |
| 37 | 37.1 | 62/ M | MHS | unknown | Asthma | yes (similar) | yes  (mild) |  |
| 38 | 38.1 | 37/ M | MHS | unknown | None | yes (severe) | yes (severe) |  |
|  | 38.2 | 35/ M | MHS | c.14524G>A  p.V4842M  + intron: c.10348-6C>G p.H3449ins33aafsX54 | None | yes  (very severe) | no |  |
| 39 | 39.1 | 42/ F | MHS | c.7304G>A  (p.R2435H) | Rhin | no | yes (severe) |  |
|  | 39.2 | 38/ M | MHN | **-** | Food | yes  (mild) | yes  (mild) |  |
|  | 39.3 | 41/ M | MHS | unknown | Rhin | yes (similar) | yes  (mild) |  |
| 40 | 40.1 | 45/ F | MHS | unknown | Rhin | yes (severe) | yes (severe) |  |
|  | 40.2 | 51/ M | MHS | unknown | Rhin | yes (similar) | yes (severe) |  |
| 41 | 41.1 | 41/ M | MHS | unknown | None | yes (similar) | no |  |
| 42 | 42.1 | 44/ M | MHS | unknown | None | no | no |  |
| 43 | 43.1 | 45/ M | MHS | c.A594G  (p.L198L) | None | yes (similar) | no |  |
| 44 | 44.1 | 46/ F | MHS | unknown | None | yes (similar) | yes (similar) |  |
| 45 | 45.1 | 31/ F | MHS | unknown | Rhin | no | no |  |
|  | 45.2 | 30/ F | MHS | c.C7354G  (p.R2452G) | None | no | yes  (mild) |  |
| 46 | 46.1 | 34/ F | MHS | c.7123G>C  (p.G2375R) | None | yes (similar) | no |  |
| 47 | 47.1 | 48/ M | MHS | unknown | Rhin | yes (similar) | yes (similar) |  |
| 48 | 48.1 | 35/ F | MHS | c.8197G>T  (p.G2733C) | Asthma | yes (similar) | no |  |
| 49 | 49.1 | 36/ F | MHN | **-** | Rhin | no | no |  |
|  | 49.2 | 35/ F | MHN | **-** | None | yes (similar) | yes (severe) |  |
|  | 49.3 | 17/ M | MHN | **-** | Food | yes  (very mild) | yes  (very mild) |  |
|  | 49.4 | 42/ F | MHS | c.C1840T  (p.R614C) | Rhin | no | yes (similar) |  |
| 50 | 50.1 | 39/ F | MHN | **-** | Eczema | yes (similar) | yes (similar) |  |
|  | 50.2 | 42/ M | MHS | unknown | None | yes (similar) | yes  (mild) |  |
| 51 | 51.1 | 40/ M | MHS | c.12828_12829insGAGGGCGCGGCGGGGCTC (p.G4284_T4285insAAGLEG) | None | yes (similar) | yes (similar) |  |
|  | 51.2 | 33/ F | MHS | c.12828_12829insGAGGGCGCGGCGGGGCTC (p.G4284_T4285insAAGLEG) | None | yes  (mild) | yes  (very mild) |  |
| 52 | 52.1 | 44/ M | MHN | **-** | Rhin, Food | yes  (mild) | yes (severe) |  |
| 53 | 53.1 | 62/ M | MHN | **-** | None | yes  (mild) | yes  (mild) |  |
| 54 | 54.1 | 46/ F | MHS | c.6488G>A (p.R2163H) | Rhin, Food | yes (similar) | yes  (very severe) |  |
|  | 54.2 | 56/ M | MHN | **-** | Rhin | yes (similar) | yes  (very severe) |  |
| 55 | 55.1 | 31/ F | MHN | **-** | None | yes  (mild) | no |  |
| 56 | 56.1 | 53**/** M | MHS | c.G14545A  (p.V4849I) | Rhin | no | no |  |
| 58 | 58.1 | 24/ F | Control | **-** | Rhin, Food | yes (similar) | yes (severe) |  |
| 59 | 59.1 | 44/ F | Control | **-** | None | yes  (very mild) | no |  |
| 60 | 60.1 | 24/ F | Control | **-** | None | no | yes  (very mild) |  |
| 61 | 61.1 | 24/ M | Control | **-** | Rhin | yes  (mild) | yes  (mild) |  |
| 62 | 62.1 | 24/ F | Control | **-** | Rhin,  Asthma | yes (similar) | yes (similar) |  |
| 63 | 63.1 | 25/ M | Control | **-** | None | yes  (mild) | yes  (mild) |  |
| 64 | 64.1 | 43/ F | Control | **-** | Eczema | yes  (mild) | yes (similar) |  |

^§^IVCT, in vitro contracture test;

^ﬁ^MHS, malignant hyperthermia susceptible;

^#^MHN, malignant hyperthermia negative;

^†^Rhin, rhinitis;

^¶^Influenza or COVID infection symptoms, compared to family members with the same infection.
