# Supplementary material for: RYR1 mutations linked to malignant hyperthermia susceptibility are associated with phenotypic changes in human B-lymphocytes
Source: Front Immunol. 2026 Jul 27;17:1876709. doi: 10.3389/fimmu.2026.1876709 (PMC13453734; doi:10.3389/fimmu.2026.1876709)
Supplement: Supplementary file 4 [file Table2.docx]

Supplementary Material

**Supplementary Table 2**. Comparison of reported allergies, viral infections and reaction to vaccination in the Brazilian MHS cohort vs healthy controls.

|  |  | **MHS**  **(n=70)** | **Healthy controls (n=31)** | **Brazilian average**  **(n=2003)** (27) | ***p value**  MHS vs Brazilian average | **Benjamin-Hochberg FDR q value** (MHS vs Brazilian average) |
| --- | --- | --- | --- | --- | --- | --- |
| **Allergies** | **Overall reported allergies** | **47.1%**  (33 out of 70) | **51.6%**  (16 out of 31) | **37.3%**  (748 out of 2003) | p=0.103 | q=0.129 |
|  | **Rhinitis** | **38.6%**  (27 out of 70) | **25.8%**  (8 out of 31) | **25.9%**  (518 out of 2003) | *p=0.026 | q=0.129 |
|  | **Asthma** | **10%**  (7 out of 70) | **6.5%**  (2 out of 31) | **4.9%**  (99 out of 2003) | p=0.087 | q=0.129 |
|  | **Atopic eczema** | **2.9%**  (2 out of 70) | **12.9%**  (4 out of 31) | **9.2%**  (185 out of 2003) | p=0.085 | q=0.129 |
|  | **Food allergies** | **12.9%**  (9 out of 70) | **19.4%**  (6 out of 31) | **10.4%**  (208 out of 2003) | p=0.549 | q=0.129 |
| **‍**  **Viral infections in vaccinated individuals**  **(influenza and COVID-19)** | **Influenza virus infection** | **77.8%**  (42 out of 54) | **89.5%**  (17 out of 19) |  |  |  |
|  | **++**  **Influenza virus infection: very mild and mild symptoms** | **33.3%**  (14 out of 42) | **64.7%**  (11 out of 17) |  |  |  |
|  | **+++**  **Influenza virus infection:**  **symptoms similar to family members** | **50%**  (21 out of 42) | **29.4%**  (5 out of 17) |  |  |  |
|  | **++++**  **Influenza virus infection: severe symptoms** | **9.5%**  (4 out of 42) | **5.9%**  (1 out of 17) |  |  |  |
|  | **+++++**  **Influenza virus infection: very severe symptoms / hospital** | **7.1**%  (3 out of 42) | **0%**  0 out of 17 |  |  |  |
|  | **COVID-19 virus infection** | **57.1%**  (40 out of 70) | **77.4%**  (24 out of 31) |  |  |  |
|  | **++**  **COVID-19 virus infection:**  **very mild and mild symptoms** | **40%**  (16 out of 40) | **41.7%**  10 out of 24 |  |  |  |
|  | **+++**  **COVID-19 virus infection:**  **symptoms similar to family members** | **30%**  (12 out of 40) | **33.3%**  (8 out of 24) |  |  |  |
|  | **++++**  **COVID-19 virus infection: severe symptoms** | **22.5%**  (9 out of 40) | **16.7%**  (4 out of 24) |  |  |  |
|  | **+++++**  **COVID-19 virus infection: very severe symptoms / hospital** | **7.5%**  (3 out of 40) | **4.2%**  (1 out of 24) |  |  |  |
| **‍**  **Reaction to vaccination** | **Influenza virus vaccination^¶^** | **77%**  (54 out of 70) | **61.2%**  (19 out of 31) |  |  |  |
|  | **Reported no reactions to influenza virus vaccination** | **37%**  20 out of 54 | **42.1%**  (8 out of 19) |  |  |  |
|  | **Reported symptomatic reactions to influenza virus vaccination** | **63%**  (34 out of 54) | **57.8%**  (11 out of 19) |  |  |  |
|  | **Covid 19 virus vaccination^#^** | **100%**  (70 out of 70) | **100%**  (31 out of 31) |  |  |  |
|  | **Reported no reactions to COVID-19 virus vaccination** | **57.9%**  (40 out of 69) | **41.9%**  (13 out of 31) |  |  |  |
|  | **Reported symptomatic reactions to COVID-19 vaccination** | **42%**  (29 out of 69) | **58%**  (18 out of 31) |  |  |  |

^¶^Received 1 dose of Influenza vaccine (Butantan Institute); ^#^Received at least 2 doses from Coronavac, Pfizer, Astra-Zeneca, Jansen, or Sichuan Clover. *Fisher’s Exact test.
