# Supplementary material for: RYR1 mutations linked to malignant hyperthermia susceptibility are associated with phenotypic changes in human B-lymphocytes
Source: Front Immunol. 2026 Jul 27;17:1876709. doi: 10.3389/fimmu.2026.1876709 (PMC13453734; doi:10.3389/fimmu.2026.1876709)
Supplement: Supplementary file 5 [file Table3.docx]

Supplementary Material

**Supplementary Table 3**: Phenotypic and genotypic characteristics of Brazilian MHS and HC cohorts donating PBMC and serum samples.

| **Patient Identification N°** | **Sample N°** | **IVCT^§^ results** | **Phenotype** | ***RYR1* mutation**  **(substitution)** | **Biological**  **Material** | **Analytical Methods** |
| --- | --- | --- | --- | --- | --- | --- |
| 15.1 | MHS 1 | MHShc Contracture: Halothane: 3.6 g and 1.56 g at 2%. Caffeine: 2.92 g and 1.2 g at 2 mMol | MHS^ﬁ^ | c.6544 A>T (p.I2182P) | EBV^†^  Serum | Total Ig, CMV Serology |
| 34.1 | MHS 2 | MHShc Contracture: Halothane: 3.4 g and 3.2 g at 2%. Caffeine: 1.04 g and 2.2 g at 2 mMol | MHS | c.487 C>T ( p.R163C) | EBV  Serum  PBMC^#^ | Total Ig, CMV Serology, IgA2 ELISA, Immunophenotyping |
| 32.1 | MHS 3 | MHShc Contracture: Halothane: 2.44 g and 3.44 g at 2%. Caffeine: 1.96 g and 1.6 g at 2 mMol. | MHS | c.7358 T>C (p.I2453T) | EBV  Serum | Total Ig, CMV Serology |
| 39.4 | MHS 4 | MHShc Contracture: Halothane: 1.48 g and 0.68 g at 2%. Caffeine: 0.28 g at 2 mMol. | MHS | c.7304G>A (p.R2435H) | EBV  Serum | Total Ig, CMV Serology, Cytokine ELISA, IgA2 ELISA, Immunophenotyping |
| 3.1 | MHS 5 | MHSh Contracture: Halothane: 0.4 g at 2% | MHS | c.1840 C>T (p.R614C) | EBV  Serum  PBMC | Total Ig, CMV Serology, IgA2 ELISA, Immunophenotyping |
| Lund 2 (33) | MHS 6 | MHS – Contracture: Halothane: 7.55 g at 2%. Caffeine: 6.2 g at 2 mMol. | MHS | c.3172G>A  (p.E1058K) | EBV | no |
| Lund 4 (33) | MHS 7 | MHS – Contracture: Halothane: 4.1 g at 2%. Caffeine: 2.4 g at 2 mMol. | MHS | c.5036G>A  (p.R1679H) | EBV | no |
| Lund 10 (33) | MHS 9 | MHS – Contracture: Halothane: 7.3 g at 2%. Caffeine: 7.2 g at 2 mMol. | MHS | c.7522C>G  (p.R2508G) | EBV | no |
| 38.2 | MHS 12 | MHShc  Contracture:  Halothane: 1.24 g and 1 g at 2%. Caffeine: 0.2 g and 0.28 g at 2 mMol. | MHS | Exon: p. V4842M (Intron: H3449ins33aafs*54) | PBMC | Immunophenotyping |
| 38.3 | MHS 13 | MHShc  Contracture:  Halothane: 0.24 g at 2%. Caffeine:0.40 g at 2 mMol. | MHS | unknown | Serum  PBMC | Total Ig, Immunophenotyping |
| 49.5 | MHS 14 | MHShc Contracture: Halothane: 1.04 g and 0.88 g at 2%. Caffeine: 0.52 g and 1.04 g at 2 mMol. | MHS | c.1840 C>T (p.R614C) | PBMC | Immunophenotyping |
| 39.1 | MHS 15 | MHShc Contracture: Halothane: 2,48g and 2,44g at 2%. Caffeine: 1,8g and 1,8g at 2mMol | MHS | c.7304G>A (p.R2435H) | Serum  PBMC | Total Ig, CMV Serology, Cytokine ELISA, IgA2 ELISA, Immunophenotyping |
| 29.1 | MHS 16 | MHShc Contracture: Halothane: 2,72 g and 1,12 g at 2%. Caffeine: 0,8 g and 0,2 g at 2 mMol | MHS | c.14545G>A (p.V4849I) | Serum  PBMC | Total Ig, CMV Serology, Cytokine ELISA, IgA2 ELISA, Immunophenotyping |
| 36.1 | MHS 17 | MHShc Contracture: Halothane:  1,4 g and 2,28 g at 2%. Caffeine: 1 g and 0,2 g at 2 mMol | MHS | c. 131AG>A (p.R44H) | Serum  PBMC | Total Ig, CMV Serology, Cytokine ELISA, IgA2 ELISA |
| 46.1 | MHS 18 | MHShc Contracture: Halothane:  2,44g and 2,52g at 2%. Caffeine: 2,52g and 2g at 2mMol | MHS | c.7123G>C (p.G2375R) | Serum  PBMC | Total Ig, Cytokine ELISA, IgA2 ELISA, Immunophenotyping |
| 54.1 | MHS 19 | MHShc Contracture: Halothane:  2,78 g and 1,66 g at 2%. Caffeine: 0,28 g and 0,42 g at 2 mMol | MHS | c.6488G>A (p.R2163H) | Serum PBMC | Total Ig, Cytokine ELISA, IgA2 ELISA, Immunophenotyping |
| Lund 3 (33) | HC 1 | no | HC^±^ | - | EBV | no |
| 58.1 | HC 2 | No | HC | - | Serum EBV | Total Ig, CMV Serology |
| 65.1 | HC 3 | no | HC | - | Serum EBV | Total Ig |
| 66.1 | HC 4 | No | HC | - | Serum EBV | Total Ig, CMV Serology, IgA2 ELISA |
| 67.1 | HC 5 | No | HC | - | Serum EBV | Total Ig, CMV Serology |
| 62.1 | HC 6 | No | HC | - | Serum EBV | Total Ig, CMV Serology |
| 63.1 | HC 7 | no | HC | - | Serum EBV | Total Ig |
| 68.1 | HC 8 | No | HC | - | Serum  EBV | Total Ig, CMV Serology, IgA2 ELISA |
| 32.2 | HC 9 | No | HC | - | Serum EBV | Total Ig, CMV Serology, IgA2 ELISA |
| 69.1 | HC 10 | No | HC | - | Serum | Total Ig, CMV Serology |
| 70.1 | HC 11 | No | HC | - | Serum | Total Ig, CMV Serology |
| 71.1 | HC 14 | No | HC | - | Serum | Total Ig, CMV Serology, IgA2 ELISA |
| 72.1 | HC 18 | No | HC | - | Serum | Total Ig, CMV Serology |
| 61.1 | HC 19 | No | HC | - | Serum PBMC | Total Ig, CMV Serology, Immunophenotyping |
| 73.1 | HC 20 | No | HC | - | Serum PBMC | Total Ig, CMV Serology, Immunophenotyping |
| 74.1 | HC 21 | No | HC | - | Serum PBMC | Total Ig, IgA2 ELISA, Immunophenotyping |
| 75.1 | HC 22 | No | HC | - | Serum | Total Ig, CMV Serology, Cytokine ELISA, IgA2 ELISA, |
| 76.1 | HC 23 | No | HC | - | Serum PBMC | Total Ig, CMV Serology, Cytokine ELISA, IgA2 ELISA, Immunophenotyping |
| 64.1 | HC 24 | No | HC | - | Serum PBMC | Total Ig, CMV Serology, Cytokine ELISA, Immunophenotyping |
| 59.1 | HC 25 | No | HC | - | Serum PBMC | Total Ig, Cytokine ELISA, IgA2 ELISA, Immunophenotyping |
| 77.1 | HC 26 | No | HC | - | Serum PBMC | Total Ig, CMV Serology, Cytokine ELISA, IgA2 ELISA, Immunophenotyping |
| 78.1 | HC 27 | No | HC | - | PBMC | Immunophenotyping |
| 79.1 | HC 28 | No | HC | - | PBMC | Immunophenotyping |
| 80.1 | HC 29 | No | HC | - | Serum PBMC | Total Ig, Cytokine ELISA, IgA2 ELISA, Immunophenotyping |
| 3.3 | HC 30 | No | HC  (Relative of Family 3) | none | PBMC | Immunophenotyping |
| 39.5 | HC 31 | No | HC  (Relative of Family 39) | none | Serum PBMC | Total Ig, CMV Serology, Immunophenotyping |
| 29.5 | HC 32 | No | HC  (Relative of Family 29) | none | Serum PBMC | Total Ig, Immunophenotyping |
| 54.3 | HC 33 | No | HC  (Relative of Family 54) | none | Serum PBMC | Total Ig, Immunophenotyping |
| 36.5 | HC 34 | No | HC  (Relative of Family 36) | none | Serum | Total Ig |
| 60.1 | HC 35 | No | HC | - | Serum | Cytokine ELISA, IgA2 ELISA |

^§^IVCT, in vitro contracture test; ^ﬁ^MHS, malignant hyperthermia susceptible;

^±^HC, healthy control; ^#^PBMC, Peripheral Blood Mononuclear Cells;

^†^EBV, Epstein Barr Virus.
