# Supplementary material for: RYR1 mutations linked to malignant hyperthermia susceptibility are associated with phenotypic changes in human B-lymphocytes
Source: Front Immunol. 2026 Jul 27;17:1876709. doi: 10.3389/fimmu.2026.1876709 (PMC13453734; doi:10.3389/fimmu.2026.1876709)
Supplement: Supplementary file 6 [file Table4.docx]

Supplementary Material

**Supplementary Table 4**: Allergy status and immune markers at the individual level.

|  | | | **PBMC and serum** | | **EBV cells** | |
| --- | --- | --- | --- | --- | --- | --- |
| **Sample N°** | **RyR1 mutation** | **Allergy** | **CD86 and CD40 normalized surface expression (median MFI)** | **IL-4**  **IL-6**  **IL-10**  **(pg/ml)** | **4-cmc EC_50_**  **µM** | **CD86 by caffeine at 48h (fold change to day 0)** |
| MHS 1 | p.I2182P | Rhin, Eczema | n.t. | n.t. | 590.3±94.8 | 7.7 ± 2.3 |
| MHS 2 | p.R163C | None | CD86: 770.0  CD40: 108.3 | n.t. | 585.6±47.6 | 2.0 ± 0.9 |
| MHS 3 | p.I2453T | Food | n.t. | n.t. | 576.1±93.5 | 13.4 ± 0.5 |
| MHS 5 | p.R614C | None | CD86: 764  CD40: 108.2 | n.t. | 540.3±24.2 | 2.7 ± 0.5 |
| MHS 6 | p.E1058K | None | n.t. | n.t. | 550±28.3 | 2.1 ± 0.6 |
| MHS 7 | p.R1679H | None | n.t. | n.t. | 480.9±65.3 | 3.0 ± 0. 8 |
| MHS 9 | p.R2508G | None | n.t. | n.t. | 501.3±39.3 | 8.3 ± 2.7 |
| MHS 12 | Exon: p. V4842M (Intron: H3449ins33aafs*54) | None | CD86: 1321  CD40: 95.5 | n.t. | n.t. | n.t. |
| MHS 15 | p.R2435H | Rhin | CD86: 1521  CD40: 111.2 | IL-4: 4.5  IL-6: 2.6  IL:10: 5.6 | n.t. | n.t. |
| MHS 16 | p.V4849I | Rhin, Food | CD86: 998  CD40: 153 | IL-4: 1.8  IL-6: 3.5  IL-10:1.1 | n.t. | n.t. |
| MHS 17 | p.R44H | None | CD86: 929  CD40: 153 | IL-4: 2.9  IL-6: 1.8  IL-10: 1.8 | n.t. | n.t. |
| MHS 18 | p.G2375R | None | CD86: 1009  CD40: 119.9 | IL-4: 1.6  IL-6: 0.7  IL-10: 0.6 | n.t. | n.t. |
| MHS 19 | p.R2163H | Rhin, Food | CD86: 837  CD40: 118.6 | IL-4: 1.6  IL-6: 0.0  IL-10: 0.5 | n.t. | n.t. |
| HC 1 | - | none | n.t. | n.t. | 717.8±65.0 | 1.30 ± 0.2 |
| HC 2 | - | Rhin, Food | n.t. | n.t. | 563±24.4 | 2.9 ± 1.3 |
| HC 6 | - | Rhin, Asthma | n.t. | n.t. | 605.9±25.1 | 0.9 ± 0.2 |
| HC 7 | - | None | n.t. | n.t. | 675.8±41.1 | 1.3 ± 0.3 |
| HC 9 | - | None | n.t. | n.t. | 671.8±56.8 | 2.8 ± 0.5 |
| HC 19 | - | Rhin | CD86: 714  CD40: 99.4 | n.t. | nt. | n.t. |
| HC 24 | - | Eczema | CD86: 712  CD40: 86.6 | IL-4: 0.7  IL-6: 0.2  IL-10: 0.0 | n.t. | n.t. |
| HC 25 | - | None | CD86: 727  CD40: 88.4 | IL-4: 0.9  IL-6: 0.0  IL-10: 0.0 | n.t. | n.t. |
| HC 35 | - | None | CD86: 882  CD40: 104.4 | IL-4: 2.2  IL-6: 0.6  IL-10: 1.2 | n.t. | n.t. |

n.t., not tested; MHS, malignant hyperthermia susceptible; HC, healthy control.
