# Supplementary material for: RYR1 mutations linked to malignant hyperthermia susceptibility are associated with phenotypic changes in human B-lymphocytes
Source: Front Immunol. 2026 Jul 27;17:1876709. doi: 10.3389/fimmu.2026.1876709 (PMC13453734; doi:10.3389/fimmu.2026.1876709)
Supplement: Supplementary file 7 [file Table5.docx]

Supplementary Material

**Supplementary Table 5:** Cytokine standards, capture antibodies, detection antibodies and commercial kits used for cytokine detection in ELISA assays.

| **Name** | **Supplier** | **Catalog Number** |
| --- | --- | --- |
| Human IL-2 | BioLegend | 79040 |
| Human IL-4 | BioLegend | 79022 |
| Human IL-6 | BioLegend | 79028 |
| Human IL-10 | BioLegend | 79031 |
| Human IL-12 (p70) | BioLegend | 79037 |
| Human IL-1α | BioLegend | 78155 |
| Human IL-1β | BioLegend | 78164 |
| Human TNF-α | BioLegend | 79019 |
| Anti-human IL-2 | BioLegend | 500310 |
| Anti-human IL-4 | BioLegend | 500702 |
| Anti -human IL-6 | BioLegend | 501102 |
| Anti -human IL-10 | BioLegend | 506802 |
| Anti -human IL-12 (p70) | BioLegend | 511002 |
| Anti -human IL-1α | BioLegend | 500104 |
| Anti -human IL-1β | BioLegend | 511602 |
| Anti -human TNF-α | BioLegend | 502802 |
| Biotin anti-human IL-2 | BioLegend | 517605 |
| Biotin anti-human IL-4 | BioLegend | 500803 |
| Biotin anti-human IL-6 | BioLegend | 501202 |
| Biotin anti-human IL-10 | BioLegend | 501502 |
| Biotin anti-human IL-12/IL-23 p40 | BioLegend | 508801 |
| Biotin anti-human IL-1α | BioLegend | 531304 |
| Biotin anti-human IL-1β | BioLegend | 511704 |
| Biotin anti-human TNF-α | BioLegend | 502903 |
| Human IL-4 ELISA kit high sensitivity | Abcam | Ab46063 |
| Human IL-6 ELISA kit high sensitivity | Abcam | Ab46042 |
| Human IL-10 ELISA kit high sensitivity | Abcam | Ab46059 |
| Human BAFF ELISA kit | Abcam | Ab119579 |
