# Supplementary material for: RYR1 mutations linked to malignant hyperthermia susceptibility are associated with phenotypic changes in human B-lymphocytes
Source: Front Immunol. 2026 Jul 27;17:1876709. doi: 10.3389/fimmu.2026.1876709 (PMC13453734; doi:10.3389/fimmu.2026.1876709)
Supplement: Supplementary file 8 [file Table6.docx]

Supplementary Material

**Supplementary Table 6**: Viability kit and antibodies against surface antigens used in flow cytometry

| **Name** | **Supplier** | **Catalog Number** | **Clone** |
| --- | --- | --- | --- |
| Invitrogen™  LIVE/DEAD™ Fixable Blue Dead Cell Stain Kit | ThermoFisher | L23105 | - |
| UltraComp eBeads™ Compensation Beads | ThermoFisher | 01-2222-42 | - |
| IgA Antibody, anti-human, APC | Miltenyi Biotec | 130-113-472 | IS11-8E10 |
| BD Horizon™ BUV395 Mouse Anti-Human IgD | BD Biosciences | 563813 | IA6-2 |
| BD Pharmingen™ PE-Cy™5 Mouse Anti-Human IgM | BD Biosciences | 551079 | G20-127 |
| BD Horizon™ BV480 Mouse Anti-Human CD3 | BD Biosciences | 566105 | UCHT1 |
| BD OptiBuild™ BUV496 Mouse Anti-Human CD86 | BD Biosciences | 749895 | BU63 |
| BD OptiBuild™ BUV563 Mouse Anti-Human CD21 | BD Biosciences | 748728 | 1048 |
| BD OptiBuild™ BUV661 Mouse Anti-Human IgG | BD Biosciences | 741639 | G18-145 |
| BD OptiBuild™ BUV737 Mouse Anti-Human CD10 | BD Biosciences | 748730 | MEM-78 |
| BD OptiBuild™ BUV805 Mouse Anti-Human CD40 | BD Biosciences | 742028 | 5C3 |
| Alexa Fluor® 700 anti-human CD27 | BioLegend | 356416 | O323 |
| APC/Fire™ 750 anti-human CD24 | BioLegend | 311140 | ML5 |
| Brilliant Violet 421™ anti-human CD19 | BioLegend | 302234 | HIB19 |
| Brilliant Violet 510™ anti-human HLA-DR | BioLegend | 307646 | L243 |
| Brilliant Violet 570™ anti-human CD14 | BioLegend | 301832 | M5E2 |
| Brilliant Violet 605™ anti-human CD20 | BioLegend | 302334 | 2H7 |
| Brilliant Violet 650™ anti-human CD38 | BioLegend | 303562 | HIT2 |
| Brilliant Violet 711™ anti-human CD83 | BioLegend | 305333 | HB15e |
| Brilliant Violet 750™ anti-human CD45 | BioLegend | 368542 | HI30 |
| Brilliant Violet 785™ anti-human CD39 | BioLegend | 328240 | A1 |
| FITC anti-human CD319 (CRACC) | BioLegend | 331818 | 162.1 |
| APC/Fire^TM^ 810 anti-human CD274 (PD-L1) | BioLegend | 374516 | BC96 |
| PE/Cyanine7 anti-human CD268 (BAFF-R) | BioLegend | 316920 | 11C1 |
| PE/Dazzle™ 594 anti-human CD56 (NCAM) | BioLegend | 362544 | HCD56 |
| PerCP/Cyanine5.5 anti-human CD85j (ILT2) | BioLegend | 333714 | GHI/75 |
| Spark Blue™ 574 anti-human CD71 | BioLegend | 334148 | CY1G4 |
